# Deletion of histone demethylase *Lsd1* (Kdm1a) during retinal development leads to defects in retinal function and structure

**DOI:** 10.3389/fncel.2023.1104592

**Published:** 2023-02-10

**Authors:** Salma Ferdous, Debresha A. Shelton, Tatiana E. Getz, Micah A. Chrenek, Nancy L’Hernault, Jana T. Sellers, Vivian R. Summers, P. Michael Iuvone, Jeremy M. Boss, Jeffrey H. Boatright, John M. Nickerson

**Affiliations:** ^1^Department of Ophthalmology, Emory University, Atlanta, GA, United States; ^2^Department of Microbiology and Immunology, Emory University, Atlanta, GA, United States; ^3^Atlanta Veterans Administration Center for Visual and Neurocognitive Rehabilitation, Decatur, GA, United States

**Keywords:** retina, *Lsd1*, Kdm1a, neuroepigenetics, retinal degeneration, neurodegeneration

## Abstract

**Purpose:**

The purpose of this study was to investigate the role of Lysine specific demethylase 1 (*Lsd1*) in murine retinal development. LSD1 is a histone demethylase that can demethylate mono- and di-methyl groups on H3K4 and H3K9. Using Chx10-Cre and Rho-iCre75 driver lines, we generated novel transgenic mouse lines to delete *Lsd1* in most retinal progenitor cells or specifically in rod photoreceptors. We hypothesize that *Lsd1* deletion will cause global morphological and functional defects due to its importance in neuronal development.

**Methods:**

We tested the retinal function of young adult mice by electroretinogram (ERG) and assessed retinal morphology by *in vivo* imaging by fundus photography and SD-OCT. Afterward, eyes were enucleated, fixed, and sectioned for subsequent hematoxylin and eosin (H&E) or immunofluorescence staining. Other eyes were plastic fixed and sectioned for electron microscopy.

**Results:**

In adult Chx10-Cre Lsd1^fl/fl^ mice, we observed a marked reduction in a-, b-, and c-wave amplitudes in scotopic conditions compared to age-matched control mice. Photopic and flicker ERG waveforms were even more sharply reduced. Modest reductions in total retinal thickness and outer nuclear layer (ONL) thickness were observed in SD-OCT and H&E images. Lastly, electron microscopy revealed significantly shorter inner and outer segments and immunofluorescence showed modest reductions in specific cell type populations. We did not observe any obvious functional or morphological defects in the adult Rho-iCre75 Lsd1^fl/fl^ animals.

**Conclusion:**

*Lsd1* is necessary for neuronal development in the retina. Adult Chx10-Cre Lsd1^fl/fl^ mice show impaired retinal function and morphology. These effects were fully manifested in young adults (P30), suggesting that *Lsd1* affects early retinal development in mice.

## Introduction

Lysine specific demethylase 1 (*Lsd1*) can demethylate mono- and di- methyl groups on specific lysine positions on Histone H3 and H4, such as H3K4 (Shi et al., 2004), H3K9 (Laurent et al., 2015), and H4K20 (Wang et al., 2015), as well as demethylate non-histone proteins (Gu et al., 2020). Although it is ubiquitously expressed throughout the body, *Lsd1* has an important role in the development of neurons, particularly due to a neuron-specific isoform, neuroLsd1 (nLsd1) (Zibetti et al., 2010; Toffolo et al., 2014; Rusconi et al., 2016a). *Lsd1* promotes neurite growth and branching (Zibetti et al., 2010) and plays a role in spatial learning and long-term memory formation (Rusconi et al., 2016b). Dysregulation of *Lsd1* in animal models causes a variety of neuronal specific abnormalities, including reduced cell proliferation in the hippocampal dentate gyri (Sun et al., 2010), abnormal development of pyramidal cortical neurons (Fuentes et al., 2012), paralysis related to degeneration of the hippocampus and cortex (Christopher et al., 2017), and an anxiety-like emotional behavior (Rusconi et al., 2016b). In humans, mutations in *LSD1* have been associated with a wide array of neurodevelopmental, psychiatric, and addiction disorders (Collins et al., 2019). Human patients with dominant missense mutations in *LSD1* have neurodevelopmental delays and craniofacial abnormalities (Chong et al., 2016; Pilotto et al., 2016) and features of KBG syndrome (OMIM #148050) and Kabuki syndrome (OMIM #147920) (Tunovic et al., 2014).

*Lsd1* is in the top 2% of evolutionarily constrained genes, which are genes that exhibit no sequence changes among widely diverse species (Samocha et al., 2014). Consequently, global homozygous deletion of *Lsd1* in mice results in embryonic lethality at embryonic day 9.5 (E9.5), likely due to cardiac problems, whereas heterozygous deletion has no profound consequences (Wang et al., 2007). During normal retinal development, *Lsd1* is expressed in all retinal progenitor cells (RPCs), and in most mature retinal neurons after development is complete (Ferdous et al., 2019). *Lsd1* is also expressed in all other major ocular structures such as the cornea, lens, and retinal pigmented epithelium (RPE) (Ferdous et al., 2019). Based on the importance of *Lsd1* in proper brain neuronal development and function as well as its ubiquitous expression in retinal neurons during and after development, we hypothesize that *Lsd1* is required for the development, maintenance, and function of the retina. The deletion of *Lsd1* could result in: (1) Retinal degeneration due to retinal progenitor cells being unable to proliferate and/or cells undergoing cell death due to aberrant epigenomic regulation; or (2) Improper differentiation of retinal progenitor cells into mature retinal neurons leading to an over- or under-representation of certain neuronal populations, specifically photoreceptors. This hypothesis is based on work done by Popova et al. (2016) in which pharmacological inhibition of LSD1 in retinal explants inhibited proper rod photoreceptor development *via* misexpression of the Notch/Hes1 pathway (Popova et al., 2016). This group then went on to discover that pharmacological inhibition of LSD1 in the rd10 mouse prevented rod photoreceptor death and improved retinal function, indicating that LSD1 inhibitors may be a viable option for treating retinal degeneration (Popova et al., 2021).

To test our hypotheses, we used the Cre-Lox system (Nagy, 2000) to delete *Lsd1* in most retinal progenitor cells using the Chx10-Cre driver mouse line (Rowan and Cepko, 2004). The expression of Chx10, also known as Vsx2, is found at embryonic stages in the mouse neuroblast; however, expression becomes increasingly restricted to the inner nuclear layer (INL) until ultimately becoming absent in most post-mitotic retinal cells except bipolar cells and some Muller glial cells in mice (Liu et al., 1994; Rowan and Cepko, 2004). Afterward, we tested juvenile P30 adult mice for retinal function (ERGs), examined *in vivo* morphology (fundus and SD-OCT), and conducted post-mortem morphology [hematoxylin and eosin (H&E) staining, immunofluorescence staining, and electron microscopy] to understand the effects of *Lsd1* deficiency. We found substantial functional abnormalities and losses in ERGs but comparatively modest structural changes at the microscopic level. We also tested whether deletion of *Lsd1* in a rod-specific manner using the Rho-iCre75 mouse line would have any effect on proper rod development or function; however, those animals did not show signs of an obvious phenotype.

## Materials and methods

### Animal studies

Mouse housing, experiments, and handling were approved by the Emory University Institutional Animal Care and Use Committee. The studies were conducted in compliance with the Association for Research in Vision and Ophthalmology (ARVO) guidelines and principles of the Association for Assessment and Accreditation of Laboratory Animal Care (AAALAC). Mice were maintained on a 12-h light/dark cycle at 22°C, with standard mouse chow (Lab Diet 5001; PMI Nutrition Inc., LLC, Brentwood, MO, USA) and water was provided *ad libitum*. Mice were managed and housed by the Emory University Division of Animal Resources. Roughly equal number of male and female mice were used in all experiments. Adult mice were euthanized using CO_2_ gas asphyxiation for 5 min followed by cervical dislocation.

### Breeding scheme

Chx10-Cre mice (JAX Stock #005105) were bred with Lsd1^fl/fl^ mice (Wang et al., 2007) gifted from Dr. Jeremy Boss at Emory University (Wang et al., 2007; Haines et al., 2018). This breeding scheme produced litters that were approximately 50% Lsd1^fl/fl^ (controls) and 50% Chx10-Cre Lsd1^fl/fl^ (experimental). These mice were produced to specifically delete *Lsd1* from most retinal progenitor cells during development. The Chx10-Cre control animals were bred separately. Additionally, Rho-iCre75 mice (JAX Stock #015850) (Li et al., 2005) were bred with Lsd1^fl/fl^ animals to produce litters that were approximately 50% Lsd1^fl/fl^ (controls) and 50% Rho-iCre75 Lsd1^fl/fl^ (experimental). These mice were produced to specifically delete *Lsd1* in rod photoreceptors. All Chx10-Cre related experiments were conducted at ∼P30 whereas Rho-iCre75 related experiments were conducted at ∼P60. All mouse lines were on a C57BL/6J background to eliminate any potential genetic confounding factors. Genotyping was performed with polymerase chain reaction (PCR). Results from genotyping for Cre recombinase were hidden from the experimental biologists until after *in vivo* experiments were complete and samples were collected to remove any possible implicit bias.

### Electroretinograms

Mice were dark-adapted overnight the day before ERGs were performed (Mazzoni et al., 2019) and experiments were conducted in dim red light conditions. Each mouse was anesthetized using intraperitoneal (IP) injections of 100 mg/kg ketamine and 15 mg/kg xylazine (ketamine; KetaVed from Patterson Veterinary, Greeley, CO, USA; xylazine from Patterson Veterinary, Greeley, CO, USA).

Once anesthetized, proparacaine (1%; Akorn Inc.) and tropicamide (1%; Akorn Inc.) eye drops were administered to reduce eye sensitivity and dilate the pupils. Mice were placed on a heating pad (39°C) under dim red light provided by the overhead lamp of the Diagnosys Celeris ERG apparatus (Diagnosys, LLC, Lowell, MA, USA). The light-guided electrodes were placed in contact with individual eyes, and the corneal electrode for the contralateral eye acted as the reference electrode. Full-field ERGs were recorded for the scotopic condition (stimulus intensities: 0.001, 0.005, 0.01, 0.1, and 1 cd s/m^2^ with a flash duration of 4 milliseconds). Signals were collected for 0.3 sec after each step to test for scotopic a- and b-wave function. To test for the c-wave, a flash intensity of 10 cd s/m^2^ was used and signal was collected for 5 sec. The c-wave was measured from baseline to the peak of the waveform. After scotopic data were captured and analyzed, mice were light-adapted for 10 min, and then full-field ERGs were recorded for the photopic conditions (stimulus intensities: 3 and 10 cd s/m^2^) to capture photopic a- and b-waves, as well as cone flicker responses at 10 Hz. After recording, each mouse was placed in its home cage on top of a heating pad (39°C) to recover from anesthesia.

### *In vivo* ocular imaging

Mice were anesthetized using IP injections of ketamine and xylazine, as described above. Once anesthetized, proparacaine and tropicamide eye drops were administered, as described above. A MICRON^®^ IV Spectral Domain Optical Coherence Tomography (SD-OCT) system with a fundus camera (Phoenix Research Labs, Pleasanton, CA, USA) was used to obtain both fundus photos and OCT images of both eyes. Images were obtained after clear visualization of the fundus with a centered optic nerve. Circular scans approximately 100 microns from the optic nerve head were taken, and fifty scans were averaged. The OCT images were analyzed for both total retinal thickness and photoreceptor layer thickness using Photoshop CS6 (Adobe Systems Inc., San Jose, CA, USA) by an individual who was masked to sample identity. The number of pixels were converted into micrometers by multiplying by a conversion factor (1 pixel = 1.3 microns).

### Immunoblotting

Immunoblot experiments were performed as previously described (Ferdous et al., 2019). Briefly, two dissected retinas were collected from each sample. Protein was extracted using mechanical shearing of the tissue by a QIAGEN TissueLyser in a solution of radioimmunoprecipitation (RIPA) buffer containing protease inhibitors (completed mini protein inhibitor catalog #118361530001) and phosphatase inhibitors (PhosSTOP EASypack #04906845001). Protein concentration of the supernatant was determined using a Pierce Bicinchoninic Acid (BCA) Assay28 and absorbance was measured at 562 nm using a Synergy H1 Hybrid Plate Reader (BioTek). After protein quantification, samples were diluted to a protein concentration of 0.8 mg/mL and immediately before electrophoresis samples were heated for 5 min at 95°C in a thermocycler. Samples were run on a pre-cast Criterion gel (BioRad TGX Stain Free Gel 4–15% Catalog #567-1083) as well as 10 mL of a molecular weight ladder (Bio-Rad Catalog #1610376) and run at 100 V for 90 min. Samples were transferred for 7 min onto PVDF blotting membrane using *Trans*-blot turbo pack (Bio-Rad Catalog #170-4157) and *Trans*-blot Turbo Transfer System (Bio-Rad). Membranes were blocked for 2 hrs at room temperature in 5% (W/V) instant non-fat dry milk (Quality Biological Catalog #A614-1005) in TBST [Tris buffered saline (TBS) (Bio-Rad #1706435) with 0.1% (V/V) Tween 20 (Fisher Scientific BP337-100)]. Afterward, primary antibodies, anti-LSD1 (Abcam 129195 [1:1,000]) and anti-GAPDH (GeneTex GTX627408 [1:1,000]), were diluted with 5% milk in TBST, and membranes were incubated overnight on a 4°C shaker. The membrane was washed three times for 5 min each using TBST. HRP conjugated secondary antibodies, mouse anti-rabbit HRP (Santa Cruz sc-2357 [1:5,000]) and goat anti-mouse HRP (Abcam ab7068 [1:5,000]), were diluted with 5% milk in TBST, and membranes were incubated for 1–2 hrs at room temperature on a shaker. The membrane was washed three times for 5 min each using TBST. A total of 10 mL of Luminata Crescendo Western HRP substrate (EMD Millipore Catalog #WBLUR0500) was applied to the membrane for 5 min. The membrane was imaged in chemiluminescence mode using the MP ChemiDoc Imaging System (Bio-Rad). Exposure times varied from 30 to 180 sec. In order to re-probe the same membrane with multiple antibodies, after imaging, 10 mL of Restore western blot stripping buffer (Thermo Scientific Catalog #21059) was applied to the blot for 10 min, the blot was washed for 5 min using TBST, and then blocked with 5% milk (W/V) in TBST and incubated with the appropriate primary and secondary antibody as described above.

### Ocular sectioning and histology

Eyes were enucleated and processed for histology by a freeze substitution method in 10 mL of dry-ice chilled 97% methanol + 3% acetic acid for 4 days at −80°C (Sun et al., 2015). Afterward, samples were exchanged for 20 min in each of the following solutions (100% ethanol twice, followed by 100% xylene twice) at room temperature and then embedded in paraffin. A total of 5-micron sagittal plane sections were cut on a microtome with a fresh blade, and sections containing the optic nerve and the center of the cornea were selected for further staining to ensure consistency across all samples. Sections were stained with (H&E) to visualize the retinal morphology. Nuclei in the outer nuclear layer (ONL), INL, and retinal ganglion cell layer (RGCL) were counted manually by an individual who was masked to sample identity. Only nuclei within a 100-micron region were counted using Photoshop CS6 at regularly spaced intervals of 500 microns apart from the optic nerve in both the inferior and superior directions. For retinal arc length, Photoshop CS6 was used to measure the distance along the retina between the inferior and superior most peripheral retina.

### Immunofluorescence

Antibody staining was performed on eyes that were enucleated and processed by a freeze substitution method in 10 mL of dry-ice chilled 97% methanol + 3% acetic acid for 4 days at −80°C (Sun et al., 2015) and embedded in paraffin as described above. Afterward 5-micron sections were cut, and slides were soaked for 2 min each in five steps of xylene, an ethanol rehydration series (100, 90, 80, 70, 60, and 50%), and TBS (Corning 46-012-CM). A Sequenza staining system (Thermo Scientific 73310017, 72110017) was used for immunostaining the slides. Slides were incubated at room temperature (RT; ∼23°C) for 30 min in blocking buffer [2.5% normal donkey serum in TBS (Corning 46-012-CM with 0.01% NaAzide)]. Slides were stained for 1 hr at RT, washed twice for 5 min each with TBST (TBS + 0.1% Tween-20; Biorad 1706531), incubated with secondary antibody for RT for 1 hr, washed twice for 5 min each with TBST, counterstained with 2.5 μM Hoechst 33342 in TBS for 10 min, and rinsed once with TBS. Vectashield Vibrance (Vector Labs H-1700) was used to mount the coverslip, and the sections were imaged using an A1R confocal on a Nikon Ti2 microscope. All primary and secondary antibodies used for this study are listed in Table 1.

**TABLE 1:** Antibody characteristics and sources.

Antibody	Antibody type	Species	Company and catalog information	Concentration	Cell type
Anti-Calbindin D28K	Primary antibody	Anti-mouse	Santa Cruz sc-365360 conjugated AF647	1:200	B type horizontal cells, subset of AC, and RGC
Anti-CARR	Primary antibody	Anti-rabbit	Primary–Millipore AB15282	1:200	Cone photoreceptors
Anti-LSD1	Primary antibody	Anti-rabbit	Abcam ab129195	1:100	
Anti-PKCalpha	Primary antibody	Anti-rabbit	Santa Cruz sc-208	1:1,000	Rod BC, subset of AC, and RGC
Anti-RBPMS	Primary antibody	Anti-guinea pig	Millipore ABN1376	1:100	RGCs
Anti-Rhodopsin	Primary antibody	Anti-mouse	Santa Cruz sc-57433 conjugated AF647	1:500	Rods
Anti-Calretinin	Primary antibody	Anti-rabbit	Santa Cruz sc-365956 conjugated AF594	1:500	Subset of AC, HC
Anti-Ribeye	Primary antibody	Anti-mouse	Santa Cruz sc17759	1:200	Photoreceptor ribbon synapses
Anti-Bassoon	Primary antibody	Anti-mouse	Enzo Synaptic Systems #141004	1:200	Photoreceptor ribbon synapses
Anti-Vimentin	Primary antibody	Anti-goat	Santa Cruz sc-7557	1:200	Muller glia
Anti-GFAP	Primary antibody	Anti-rabbit	Dako Z0334	1:200	Muller glia
IgG AF647	Secondary antibody	Goat anti-guinea pig	Life Technologies A21450	1:1,000	
IgG-AF568	Secondary antibody	Donkey anti-rabbit	Thermo Fisher A10042	1:1,000	
IgG-AF647	Secondary antibody	Donkey anti-rabbit	Life Technologies A32795	1:1,000	
IgG-AF647	Secondary antibody	Rabbit anti-goat	Life Technologies A21446	1:1,000	
TUNEL	N/A	N/A	Promega DeadEnd TUNEL Fluorometric kit–G3250		

### TUNEL

The manufacturer instructions for the Promega DeadEnd TUNEL Fluorometric kit (Promega G3250) were followed. In brief, tissue sections were deparaffinized in 5 steps of xylene for 2 min each. The tissue sections were then rehydrated in a graded ethanol series (100, 90, 80, 70, 60, and 50%) for 2 min each. The slides were then washed for 5 min in PBS (Corning 46-013-CM) and mounted in the Sequenza system. Sections were incubated for 15 min in Z-fix (Anatech, Fisher Scientific NC935141), washed twice in PBS for 5 min each, incubated in Proteinase K solution for 8 min, washed with PBS for 5 min, fixed with Z-fix for 5 min, washed with PBS for 5 min, incubated with rTDT enzyme and nucleotide mix in equilibration buffer for 2 h, washed with 2× SSC for 5 min, counterstained with 2.5 mHoechst 33342 in TBS for 10 min, and rinsed with TBS for 5 min. Coverslips were then mounted using VectaShield Vibrance and imaged using an A1R confocal on a Nikon Ti2 microscope.

### Electron microscopy

Eyes are enucleated and fixed in 2.5% glutaraldehyde in 0.1 M Cacodylate buffer for 2 h at RT and then overnight at 4°C. Afterward, tissue was washed in 0.1 M sodium cacodylate buffer for 15 min before being post-fixed in 1% OsO_4_ in 0.1 M sodium cacodylate buffer for 2 h at RT. The tissue was washed in deionized water for 10 min and then dehydrated in a graded ethanol series (35, 50, 70, 95, and 100% twice) for 15 min each. The tissue was washed in propylene oxide twice for 15 min before being placed overnight in a 1:1 mixture of propylene oxide: LX 112 embedding resin (Ladd Research, Williston, VT, USA) overnight. The following day, the tissue was placed in pure resin within a vacuum desiccator for 3–4 h. Finally, the tissue was embedded in fresh resin and placed at 60°C for 2 days to polymerize. A Lecia UCT was used to cut 1-micron thick sections, which were stained in an aqueous solution of 1% toluidine blue and 1% sodium borate. Ultrathin sections (silver-gray) were cut from areas of interest, placed on 300 mesh copper grids and stained with 2% aqueous uranyl acetate for 30 min, before being washed in distilled water, stained with Reynold’s lead citrate for 2 min, washed and air dried. Finally, images were taken with a JEOL 100 CX-11 transmission electron microscope and photographed with a SIA L12C Peltier-cooled CCD digital camera (Scientific Instruments and Applications, Inc., Duluth, GA, USA 30096).

### Statistical analysis

Statistical analysis was conducted using Prism 8.4.2 (GraphPad Software, Inc., La Jolla, CA, USA) on Mac OS 11.6.8. All data are summarized as the mean ± standard deviation (SD), and individual statistical tests and sample sizes are listed in the figure legends. *P*-values < 0.05 were considered statistically significant. Each sample group member is an independent mouse.

## Results

**FIGURE 1:**
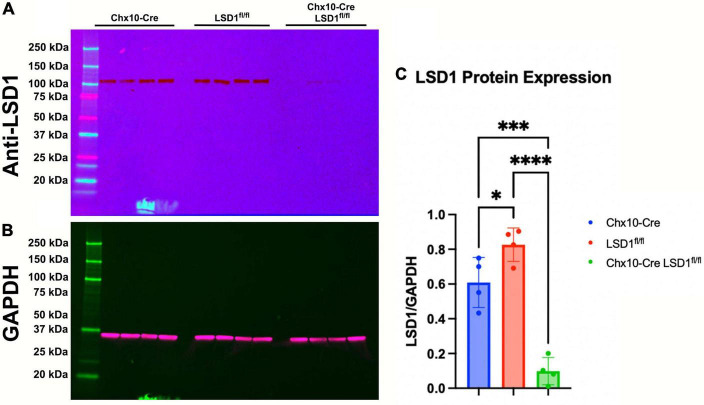
Western blot confirming LSD1 deletion in P30 retinas. Retinas from Chx10-Cre only, Lsd1^fl/fl^ only, and Chx10-Cre Lsd1^fl/fl^ were probed with a anti-LSD1 antibody [1:1,000] **(A)** and an anti-GAPDH antibody [1:1,000] **(B)** as a loading control. In the two control groups, Chx10-Cre and Lsd1^fl/fl^ only, the full length LSD1 (molecular weight: 107 kDa) was detected, with no other extraneous bands, indicating the high specificity of this antibody. Densitometric quantification showed a statistically significant decrease in protein expression in the Chx10-Cre Lsd1^fl/fl^ samples compared to both control groups **(C)**. One–Way ANOVA with Tukey’s multiple comparisons test. *Represents *p*-value < 0.05; ***represents *p*-value < 0.001; and ****represents *p*-value < 0.0001. Sample sizes: Chx10-Cre (*n* = 4), Lsd1^fl/fl^ only (*n* = 4), and Chx10-Cre Lsd1^fl/fl^ (*n* = 4). Full list of statistical results can be found in Supplementary Table 1.

The Chx10-Cre driver mouse line expresses Cre recombinase as early as E14.5 in most retinal progenitor cells (Rowan and Cepko, 2004). After breeding Chx10-Cre mice with Lsd1^fl/fl^ mice, we first tested the efficiency of Lsd1 deletion in the retina *via* western immunoblotting. Retinas were isolated from P30 mice from two control lines, Chx10-Cre only and Lsd1^fl/fl^ only to serve as negative controls that have normal Lsd1 expression and function, and one experimental group Chx10-Cre Lsd1^fl/fl^. We probed for LSD1 protein expression (Figure 1A) and used GAPDH as a loading control (Figure 1B). All eight control retinas exhibited LSD1 protein bands at the expected molecular weight of 107 kDa. Although there was some LSD1 protein expression in the four Chx10-Cre Lsd1^fl/fl^ samples, quantification *via* densitometry showed a statistically significant 86% reduction of LSD1 in the Chx10-Cre Lsd1^fl/fl^ group compared to the Chx10-Cre and Lsd1^fl/fl^ control groups (Figure 1C and Supplementary Table 1). This indicates that *Lsd1* was deleted in a high percentage of retinal cells and is consistent with known expression patterns of Chx10 in most retinoblast and mature inner retinal cells, particularly bipolar cells (Liu et al., 1994; Rowan and Cepko, 2004).

**FIGURE 2:**
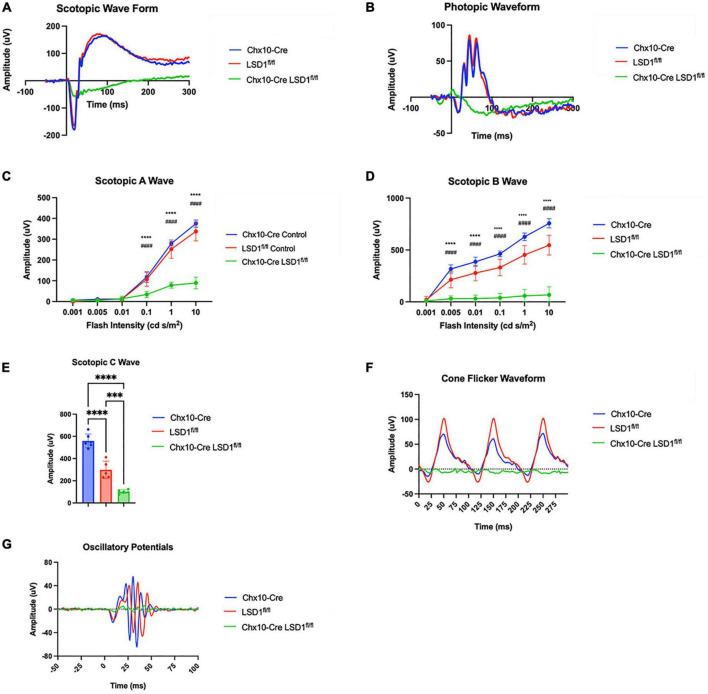
Chx10-Cre Lsd1^*fl*/*fl*^ mice have relatively flat ERG response in scotopic and photopic conditions compared to controls. Raw electroretinogram waveforms from Chx10-Cre controls, Lsd1^*fl*/*fl*^ controls and Chx10-Cre Lsd1^*fl*/*fl*^ in scotopic **(A)** and photopic **(B)** conditions after a 10 cd s/m^2^ light flash. At multiple flash intensities, both control groups show a relatively normal response for scotopic a-wave **(C)**, scotopic b-wave **(D)**, and scotopic c-wave **(E)**; however, the Chx10-Cre Lsd1^*fl*/*fl*^ mice have significant reductions in ERG response, suggesting dysfunctional photoreceptors and bipolar cells. The Chx10-Cre Lsd1^*fl*/*fl*^ mice also have profound reductions in cone flicker response at 10 Hz compared to controls **(F)** as well as reductions in oscillatory potentials **(G)**. Two–Way ANOVA with Tukey’s multiple comparisons test ***^,###^*p*-value < 0.001; ****^,####^*p*-value < 0.0001. *Symbols indicate significance between Chx10-Cre controls and Chx10-Cre Lsd1^*fl*/*fl*^; ^#^symbols indicate significance between Lsd1^*fl*/*fl*^ controls and Chx10-Cre Lsd1^*fl*/*fl*^. Samples sizes: Chx10-Cre (*n* = 6–7), Lsd1^*fl*/*fl*^ (*n* = 5), Chx10-Cre Lsd1^*fl*/*fl*^ (*n* = 4–5). Full list of statistical results can be found in Supplementary Tables 2–4.

After *Lsd1* deletion was confirmed *via* immunoblotting, adult P30 animals were tested for retinal function using full field electroretinograms (ERGs). Animals were tested in both scotopic and photopic conditions, and both a- and b-waves were measured. For both scotopic and photopic conditions, ERG waveforms in response to a 10 cd s/m^2^ light flash showed relatively normal ERG responses in the Chx10-Cre and Lsd1^fl/fl^ control animals. However, the Chx10-Cre Lsd1^fl/fl^ mice showed sharply reduced and abnormal retinal responses (Figures 2A, B). In scotopic conditions with increasing light flash intensities, we observed significant decreases in the a-wave amplitudes (∼75%) and b-wave amplitudes (∼89%) of the Chx10-Cre Lsd1^fl/fl^ animals when compared to controls, indicating possible dysfunction in the rod photoreceptors and rod bipolar cells (Figures 2C, D and Supplementary Tables 2, 3). Additionally, scotopic c-wave amplitudes were significantly reduced (Figure 2E and Supplementary Table 4), cone flicker responses were abolished, and oscillatory potentials were profoundly reduced (Figures 2F, G), indicating possible dysfunction in the RPE and abolishment of cone photoreceptor signals.

**FIGURE 3:**
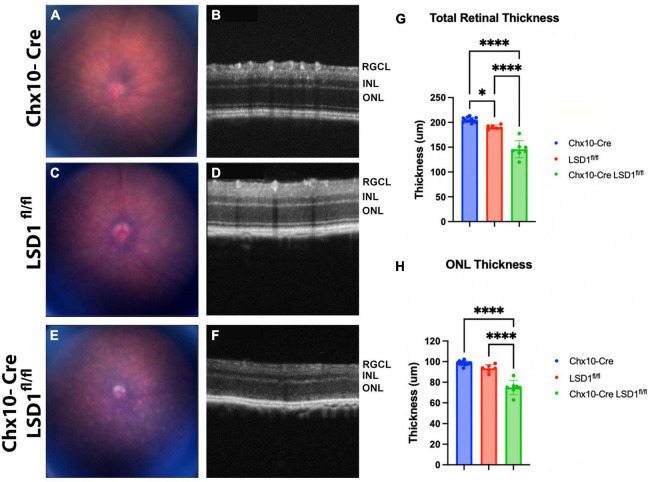
Chx10-Cre Lsd1^*fl*/*fl*^ animals show an increased mottled and speckled appearance in fundus photos **(E)** compared to both control groups **(A,C)**. In SD-OCT images, the control groups **(B,D)** have normal retinal morphology with clear, distinct layers; however, the Chx10-Cre Lsd1^*fl*/*fl*^ mice have retinas that are hazy and less distinct **(F)**. After quantification of SD-OCT images by a masked individual, the Chx10-Cre Lsd1^*fl*/*fl*^ showed significantly thinner total retinas and ONLs compared to both control groups **(G,H)**. One–Way ANOVA with Tukey’s multiple comparisons test. *P-value < 0.05; ****p-value < 0.0001. Samples sizes: Chx10-Cre (*n* = 12), Lsd1^*fl*/*fl*^ (*n* = 6), Chx10-Cre Lsd1^*fl*/*fl*^ (*n* = 7). Full list of statistical results can be found in Supplementary Tables 5, 6.

The significant retinal function defects in Chx10-Cre Lsd1^fl/fl^ mice suggest that there may be developmental abnormalities in photoreceptors and bipolar cells. To detect *in vivo* retinal morphology defects, animals were assessed at P30 with fundus photography and SD-OCT imaging. Total retinal thickness and ONL thickness were quantified by a masked individual. In the fundus photos, we observed a more mottled and speckled appearance in the Chx10-Cre Lsd1^fl/fl^ animals compared to the Chx10-Cre and Lsd1^fl/fl^ controls (Figures 3A, C, E). The SD-OCT images revealed substantial degeneration and increased hyper-reflectivity in the ONL in the Chx10-Cre Lsd1^fl/fl^ animals compared to the controls (Figures 3B, D, F). There was a statistically significant reduction in total retinal thickness (∼26% loss) and ONL thickness (∼22% loss) (Figures 3G, H and Supplementary Tables 5, 6). The significant 22% reduction in ONL thickness likely contributes in part to the 75% loss of scotopic a-wave amplitudes.

**FIGURE 4:**
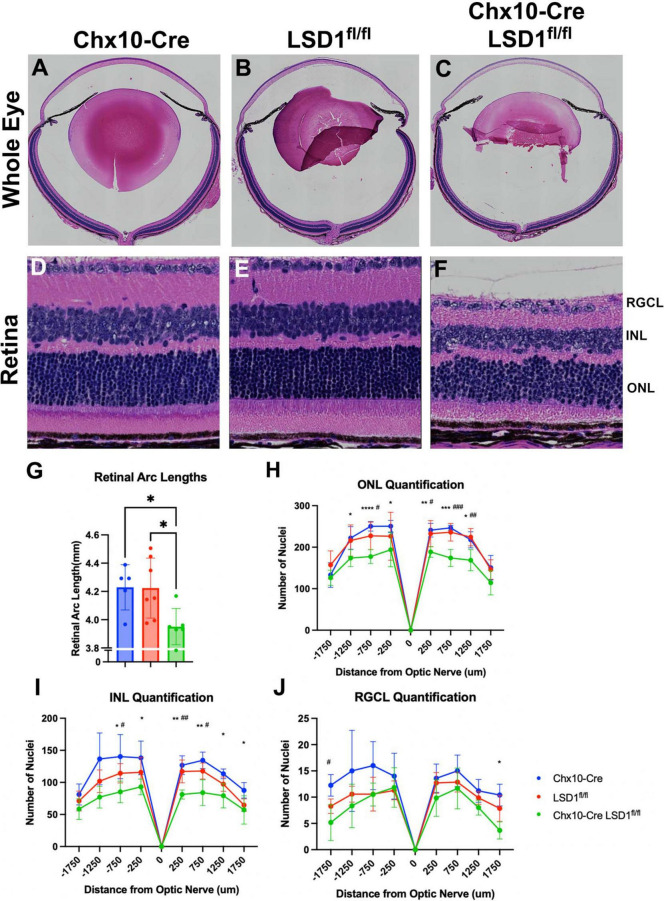
Chx10-Cre Lsd1^*fl*/*fl*^ showed modest signs of retinal thinning and irregular morphology in Hematoxylin and Eosin (H&E) staining compared to controls; however, the laminar structure and organization was present. Whole eye images **(A–C)** and high magnification retina images **(D–F)** are shown for all three groups. Chx10-Cre Lsd1^*fl*/*fl*^ mice show significant total retinal thinning and disorganized ONL and INL **(F)** compared to both control groups in panels **(D,E)**. Quantification of total retinal arc length **(G)** show significant decrease in retinal arc length in Chx10-Cre Lsd1^*fl*/*fl*^ mice compared to control groups. Quantification of nuclei in the ONL **(H)**, INL **(I)**, and RGCL **(J)** show significant decreases in cell number in ONL and INL, but not RGCL, of Lsd1^*fl*/*fl*^ mice compared to both control groups. Quantification of total retinal arc length **(J)** show significant decrease in retinal arc length in Chx10-Cre Lsd1^*fl*/*fl*^ mice compared to control groups. One–Way ANOVA with Tukey’s multiple comparisons test for retinal lengths and Two–Way ANOVA with Tukey’s multiple comparisons test for ONL, INL, and RGCL cell nuclei quantification *^,#^*p*-value < 0.05; **^,##^*p*-value < 0.01; ***^,###^*p*-value < 0.001; ****p-value < 0.0001. *Symbols indicate significance between Chx10-Cre controls and Chx10-Cre Lsd1^*fl*/*fl*^; ^#^symbols indicate significant between Lsd1^*fl*/*fl*^ controls and Chx10-Cre Lsd1^*fl*/*fl*^. Samples sizes: Chx10-Cre (*n* = 5), Lsd1^*fl*/*fl*^ (*n* = 7), Chx10-Cre Lsd1^*fl*/*fl*^ (*n* = 6). Full list of statistical results can be found in Supplementary Tables 7–10.

After *in vivo* measurements, we collected P30 eyes for post-mortem analysis. We first stained sagittal retinal sections with H&E to observe retinal morphology (Figures 4A–F). These sections showed retinal thinning similar to what was observed in SD-OCT images. Significant decreases in retinal arc lengths (∼7%) were observed in the Chx10-Cre Lsd1^fl/fl^ animals compared to controls (Figure 4G). Cell nuclei quantification of the ONL and INL showed statistically significant losses (∼19 and ∼30%, respectively) between the Chx10-Cre Lsd1^fl/fl^ group and Chx10-Cre or Lsd1^fl/fl^ control groups (Figures 4H, I and Supplementary Tables 7, 8). However, there were no statistical differences in the RGCL (Figure 4J and Supplementary Table 9). This may indicate that LSD1 protein functionality is more important for the proper development of photoreceptors and inner neurons, such as bipolar cells and horizontal cells, rather than retinal ganglion cells or displaced amacrine cells. Alternatively, it may indicate that because retinal ganglion cells and amacrine cells, along with horizontal cells, are the first cells to develop in the retina, these cell types are fully committed and differentiated before LSD1 enzyme activity is reduced in those cells by Chx10-Cre mediated deletion (Cepko et al., 1996; Bassett and Wallace, 2012).

**FIGURE 5:**
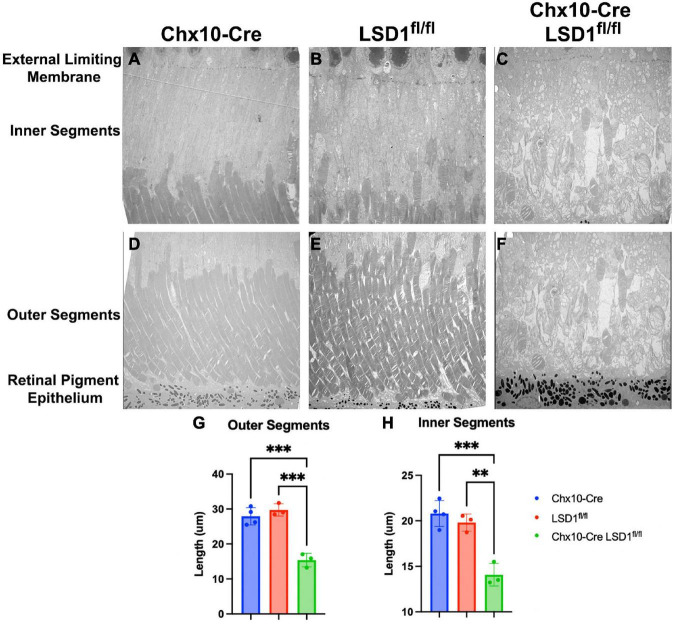
Electron microscopy of the area between RPE and external limiting membrane (ELM) show decreased lengths of inner and outer segments in Chx10-Cre Lsd1^*fl*/*fl*^ animals compared to controls. Using 1400X magnification, we observed disorganization and significant reductions of the inner and outer segments lengths **(C,F)**; however, no abnormalities were observed in the Chx10-Cre only controls **(A,D)** or the Lsd1^*fl*/*fl*^ only controls **(B,E)**. Additionally, no abnormalities, such as drusenoid like deposits or vacuoles, were observed in the ELM, RPE, or choroid in any mouse strain. Statistically significant decreases in the outer segment length **(G)** and inner segment length **(H)** were observed in Chx10-Cre Lsd1^*fl*/*fl*^ animals compared to controls. Two–Way ANOVA with Tukey’s multiple comparisons test. **Represents *p*-value < 0.01; ***represents *p*-value < 0.001. Samples sizes: Chx10-Cre (*n* = 4), Lsd1^*fl*/*fl*^ (*n* = 4), Chx10-Cre Lsd1^*fl*/*fl*^ (*n* = 5). Full list of statistical results can be found in Supplementary Tables 11, 12.

We used electron microscopy to obtain more detailed views of the abnormal morphology of the area between the RPE and the external limiting membrane (ELM). We observed substantial degeneration and disorganization of the inner and outer segments (Figures 5C, F), which may be the cause of the retinal function loss in the Chx10-Cre Lsd1^fl/fl^ mice compared to the Chx10-Cre or Lsd1^fl/fl^ controls (Figures 5A, B, D, E). We observe large vacuoles and no clear demarcation between the inner and outer segments in the Chx10-Cre Lsd1^fl/fl^ mice compared to the Chx10-Cre or Lsd1^fl/fl^ controls. On average, we found that the inner segments and outer segments of the Chx10-Cre Lsd1^fl/fl^ mice were significantly shorter compared to both control groups (∼31 and 47%, respectively, Supplementary Tables 11, 12). The average length of the outer segments in the Chx10-Cre Lsd1^fl/fl^ mice was 15.4 μm, compared to 27.9 μm for the Chx10-Cre controls and 29.7 μm for the Lsd1^fl/fl^ controls (Figure 5G). The average length of the inner segments in the Chx10-Cre Lsd1^fl/fl^ mice was 14.1 μm, compared to 20.8 μm for the Chx10-Cre controls and 19.8 μm for the Lsd1^fl/fl^ controls (Figure 5H).

**FIGURE 6:**
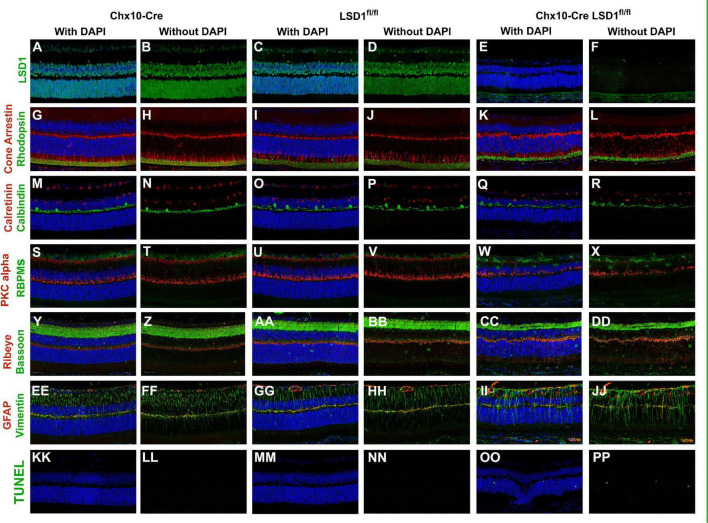
Immunofluorescence staining of Lsd1, various major cell specific markers, and TUNEL show decreased presence of specific retinal neurons and increased apoptosis in Chx10-Cre Lsd1^fl/fl^ animals compared to controls. Lsd1 protein has little to no expression in Chx10-Cre Lsd1^fl/fl^ animals [panels **(E,F)**] compared to both control groups [panels **(A–D)**]. Qualitatively, expression of photoreceptor marker cone arrestin and rhodopsin [panels **(G–L)**] are relatively consistent across the three groups. Expression of calretinin was qualitatively uniform across the three groups; however, calbindin positive cell bodies seem reduced in the Chx10-Cre Lsd1^fl/fl^ animals [panels **(Q,R)**] compared to controls [panels **(M–P)**]. RBPMS expression was qualitatively uniform across the three groups, but PKCalpha was reduced in the Chx10-Cre Lsd1^fl/fl^ animals [panels **(W,X)**] compared to controls [panels **(S–V)**]. Bassoon and Ribeye staining [panels **(CC,DD)**] seems qualitatively different in the Chx10-Cre Lsd1^fl/fl^ compared to controls [panels **(Y–BB)**]. Expression of Muller glia cell markers Vimentin and GFAP had a qualitatively increased co-localization in the Chx10-Cre Lsd1^fl/fl^ [panels **(II,JJ)**] compared to controls [panels **(EE–HH)**]. No TUNEL positive cells were observed in the control groups [panels **(KK–NN)**], but several were observed in the Chx10-Cre Lsd1^fl/fl^ animals [panels **(OO,PP)**]. Samples sizes: Chx10-Cre (*n* = 5), Lsd1^fl/fl^ (*n* = 5), Chx10-Cre Lsd1^fl/fl^ (*n* = 5).

Because of the significant decreases in cell nuclei observed in the ONL and INL, we wanted to determine whether certain cell types were specifically sensitive to Lsd1 deletion. Through immunofluorescence, we stained for LSD1, TUNEL, and major cell type specific markers across all three groups. There was little LSD1 protein expressed in the Chx10-Cre Lsd1^fl/fl^ animals compared to Chx10-Cre or Lsd1^fl/fl^ groups (Figures 6A–F), which corroborates the western blot results in Figure 1. When we examined cell-type specific markers for photoreceptors (Figures 6G–L), horizontal, amacrine (Figures 6M–R), rod bipolar cells and retinal ganglion cells (Figures 6S–X), we observed qualitative reductions in the expression of calbindin and PKCalpha in the Chx10-Cre Lsd1^fl/fl^ animals compared to controls. One interesting note is the possible mislocalization of cone cells bodies in the ONL as well as the presence of RBPMS + cells in the INL of the Chx10-Cre Lsd1^fl/fl^ mice. These cells may be displaced retinal ganglion cells, an extremely rare retinal subtype (Dräger and Olsen, 1981; Buhl and Dann, 1988; Doi et al., 1994; Nadal-Nicolás et al., 2014; Kisseleff et al., 2021); however, further validation *via* co-labeling of multiple antibodies and retrograde labeling beyond the scope of this present study are needed. We also investigated synaptic connections by co-labeling Bassoon and Ribeye, which are two components of the photoreceptors ribbon synapses (Brandstätter et al., 1999; Schmitz et al., 2000). Qualitatively, there do seem to be differences in the expression of these proteins in the Chx10-Cre Lsd1^fl/fl^ animals compared to Chx10-Cre or Lsd1^fl/fl^ groups (Figures 6Y–DD). Finally, we stained for Vimentin and GFAP to determine whether Muller glial cells were upregulated in the Chx10-Cre Lsd1^fl/fl^ animals (Figures 6EE–JJ). We observed qualitatively increased co-localization of Vimentin and GFAP in the Chx10-Cre Lsd1^fl/fl^ animals compared to Chx10-Cre or Lsd1^fl/fl^ groups. We also observed statistically significantly increases in TUNEL staining, which labels DNA breaks during apoptosis, in the Chx10-Cre Lsd1^fl/fl^ group in the ONL, consistent with ongoing photoreceptor cell death. As expected, no TUNEL staining was observed in either control group (Figures 6KK–PP and Supplementary Figure 1).

In addition to the Chx10-Cre driver line, we used a Rho-iCre75 driver to delete *Lsd1* in a rod-specific manner and tested animals at P60. In order to determine whether Lsd1 was deleted in rods, we performed antibody for LSD1 in both Lsd1^fl/fl^ control sections and Rho-iCre75 Lsd1^fl/fl^ sections (Supplementary Figure 2). LSD1 was observed in roughly all cell types in the control Lsd1^fl/fl^ retina, which agrees with our previously published work (Ferdous et al., 2019); however, there is an absence of LSD1 only in rod photoreceptors in the Rho-iCre75 Lsd1^fl/fl^ sections, indicating successful rod-specific deletion. To determine whether the deletion of *Lsd1* in rods has any effect on retinal function, we performed ERGs in scotopic conditions. There was no marked difference between the waveforms of the Lsd1^fl/fl^ control group and the Rho-iCre75 Lsd1^fl/fl^ group (Supplementary Figure 3A). There was no statistically significant difference in a-wave, b-wave, or c-wave amplitudes between the control Lsd1^fl/fl^ mice and Rho-iCre75 Lsd1^fl/fl^ mice (Supplementary Figures 3B–D). Additionally, we performed *in vivo* imaging to determine whether there were any morphological defects. Based on the fundus photographs (Supplementary Figures 4A, B) and SD-OCT photographs (Supplementary Figures 4C, D), there were no obvious signs of structural abnormalities. These *in vivo* results were corroborated with post-mortem results with H&E staining of whole eye (Supplementary Figures 5A, B) and retina (Supplementary Figures 5C, D) that showed no statistical difference in ONL nuclei counts (Supplementary Figure 5E).

## Discussion

The goal of this study was to determine the role of *Lsd1* in retinal development by genetically ablating *Lsd1* throughout the retina early during development or specifically in rods. This was achieved by using either a Chx10-Cre driver to delete *Lsd1* specifically in most RPCs or a Rho-iCre75 driver to delete *Lsd1* specifically in rod photoreceptors. To our knowledge, this is the first study to investigate the results of *in vivo* deletion of Lsd1 in the mouse retina. Overall, our results showed that the Chx10-Cre Lsd1^fl/fl^ mouse exhibited successful deletion of LSD1 protein, resulting in functional and structural defects in the mouse retina at P30. We also showed that a P60 Rho-iCre75 Lsd1^fl/fl^ mouse retina showed no obvious defects, suggesting that Lsd1 may play a more critical role in the early embryonic stages of retinal development, rather than post-natal development when many cell types are already developmentally committed and differentiated.

Previously we hypothesized that *Lsd1* ablation could result in either (1) Retinal degeneration due to RPC apoptosis or abnormalities in RPC proliferation, or (2) irregularities in the relative proportion of various mature retinal neuron subtypes due to improper RPC differentiation. Given that the current study is limited to one time point (P30), which is after retinal development is completed, we are unable to differentiate between these two possibilities; however, we consider the first scenario more likely for the following reasons. Small interfering RNA (siRNA) knockdown of LSD1 expression decreased neural stem cell proliferation in cell culture and in the hippocampal dentate gyri of adult mouse brains (Sun et al., 2010). Nestin-Cre Lsd1^fl/fl^ animals also showed a significant depletion of the neural progenitor/precursors cell population during cortical development (Zhang et al., 2014). RPCs are heterogenous in their individual transcriptome and this allows for multipotency (Trimarchi et al., 2008; Bassett and Wallace, 2012; Cepko, 2014). Under the influence of different cell fate determinants, the RPCs undergo asymmetrical cell division and become increasingly restricted and specified before committing to a particular cell fate (Cepko et al., 1996; Livesey and Cepko, 2001; Saito et al., 2003; Kechad et al., 2012). These restricted RPCs often have distinct molecular and transcriptional profiles compared to their multipotent counterparts (Blackshaw et al., 2004; Aldiri et al., 2017; Buenaventura et al., 2018; Clark et al., 2019; Shiau et al., 2021). Thus, during retinal development, the seven major and ∼130 subtypes of mature retinal cells are born in distinct, but overlapping windows of time in a stereotypical order (Young, 1985; Cepko et al., 1996; Bassett and Wallace, 2012; Shekhar et al., 2016; Tran et al., 2019; Yan et al., 2020). Therefore, we hypothesize that the loss of *Lsd1* would affect the proliferation, specification, and differentiation of the RPCs due to global abnormalities in the epigenetic environment. Given the early expression of Cre recombinase in the Chx10-Cre animals (E14.5) (Rowan and Cepko, 2004) and the consistent decreases in total retinal thickness, nuclear layer thickness and cell quantification as well as active cell death as indicated by TUNEL positive staining, we suggest that the RPC pool could be reduced in the Chx10-Cre Lsd1^fl/fl^ animals; thus, leading to the observed phenotype. Of course, there is scientific evidence of the role of Lsd1 in the differentiation of neural stem cells and neural progenitors (Fuentes et al., 2012; Reilly et al., 2015); however, differences between Lsd1 in proliferation vs. differentiation may be species specific (Hirano and Namihira, 2016). Our current results are unable to unequivocally distinguish between possible defects in RPC proliferation or RPC cell death as this will require earlier timespoints during embryonic and post-natal development. It is likely that the true answer is a combination of the two hypotheses mentioned previously.

In general, the influence of epigenetic regulation on neuronal developmental and diseases, also known as “neuroepigenetics,” is now widely recognized; histone methylation in particular has been heavily studied (Christopher et al., 2017). There are numerous examples of dysfunctional epigenetic regulation disrupting the proper development of the retina. For example, microRNAs (miRNAs), such as let-7 miR-9 and miR-125 influence the transition of retinal progenitor cells from the early to late stage (La Torre et al., 2013), whereas the polycistronic miR-183/96/182 cluster affects the proper differentiation and maintenance of cone photoreceptors (Busskamp et al., 2014; Fan et al., 2017; Xiang et al., 2017, 2022; Zhang et al., 2020). Histone modifiers, such as the demethylases JMJD3, UTX, and LSD1, provide additional regulation. For example, the loss of JMJD3 and UTX affects the proper development of inner retinal cells such as bipolar, amacrine, and horizontal cells (Iida et al., 2014; Iwagawa et al., 2020; Umutoni et al., 2020). *Lsd1* inhibition directly impacts the survival of specific retinal cells, such as RGCs and photoreceptors (Tsutsumi et al., 2016; Popova et al., 2021), and alters regulation of microRNAs, such as the miR-21-5p/NLRP12 axis, to facilitate RGC pyroptosis (Yu et al., 2022).

One interesting note is that we observed small but statistically significant differences between the Chx10-Cre and Lsd1^fl/fl^ control groups for a few parameters, including LSD1 protein expression (Figure 1C), scotopic b-waves (Figure 2D), and total retinal thickness as measured by OCT imaging (Figure 3E). Although unexpected, it is possible that the Chx10-Cre only animals may have experienced some retinal toxicity due to the presence of Cre recombinase. Although rare, there have been reports about differences in recombinase efficiency in different tissues and Cre recombinase toxicity (Loonstra et al., 2001; Hameyer et al., 2007; Naiche and Papaioannou, 2007; Lexow et al., 2013). Importantly, three different Nestin-Cre mouse strains were shown to develop hydrocephaly due to high amounts of Cre recombinase expression in neural progenitor cells (Forni et al., 2006). It is possible that the Chx10-Cre animals have slight Cre toxicity resulting in lower protein expression, overstimulation of bipolar cells, and small amounts of edema in the retina which accounts for the small, but significant differences we observe between the control groups. Overall, it does not detract from the important role of Lsd1 in proper retinal development, but it should be noted.

One limitation of the present study is the relatively crude identification of the loss of specific major cell types *via* immunofluorescence. More sophisticated methods such as RNAscope (Wang et al., 2012), or single-cell spatial technologies such as MERFISH (Chen et al., 2015; Moffitt and Zhuang, 2016), Seq-Scope (Cho et al., 2021), or SABER-FISH (Kishi et al., 2019), which has already been applied to the retina to investigate bipolar cell subtype identify, location, and birthdate (West et al., 2022), could be used to elucidate precisely which retinal subtypes are missing following *Lsd1* deletion during retinal development. Although immunofluorescence is relatively crude, it does not diminish the overall conclusions that some cell types seem to be more sensitive to *Lsd1* deletion.

Future studies will investigate how the possible epigenetic dysregulation that occurs with the loss of *Lsd1* may affect RPC development by studying different timespoints during retinal development. This can be achieved by looking at the global retinal morphology and differentiation of specific cell types during embryonic and post-natal retinal development. Additionally, it will be useful to study how the global transcriptome and epigenome are altered in these animals both during and after development through RNA-seq (Mortazavi et al., 2008; Nagalakshmi et al., 2008), ChIP-seq (Johnson et al., 2007; Robertson et al., 2007)/CUT&RUN (Skene and Henikoff, 2017), and ATAC-seq (Buenrostro et al., 2013) methods. This could lead to significant insights on the mechanistic role of *Lsd1* in the proper development and differentiation of the retina.

## Data availability statement

The original contributions presented in this study are included in the article/Supplementary material, further inquiries can be directed to the corresponding author.

## Ethics statement

The animal study was reviewed and approved by the Emory University Institutional Care and Use Committee.

## Author contributions

SF, DS, TG, JHB, and JN were involved in experimental design. SF, DS, TG, MC, NL’H, JS, and VS conducted the experiments and analyzed the data. PI, JMB, JHB, and JN provided the mouse lines and equipment. SF and JN were involved in overall study design. SF, DS, and JN wrote the manuscript. All authors discussed the results and edited the manuscript.

## Supplementary material

Supplementary Figure 1 Increased number of TUNEL positive cells in the Chx10-Cre Lsd1^*fl*/*fl*^ compared to the controls. Two–Way ANOVA with Tukey’s multiple comparisons test. ***Represents *p*-value < 0.001. Samples sizes: Chx10-Cre (*n* = 5), Lsd1^*fl*/*fl*^ (*n* = 5), and Chx10-Cre Lsd1^*fl*/*fl*^ (*n* = 5).

Supplementary Figure 2 Rho-iCre75 Lsd1^*fl*/*fl*^ mice have reduced expression of LSD1 compared to Lsd1^*fl*/*fl*^ controls. Immunofluorescence staining of LSD1 in P60 retinas showed normal LSD1 protein expression in all major cell types in the Lsd1^*fl*/*fl*^ controls; however, there is a reduction in LSD1 specifically in rod photoreceptors, but no other major cell type, in the Rho-iCre75 Lsd1^*fl*/*fl*^ mice. Samples sizes: Lsd1^*fl*/*fl*^ (*n* = 3), Rho-iCre75 Lsd1^*fl*/*fl*^ (*n* = 3).

Supplementary Figure 3 Rho-iCre75 Lsd1^*fl*/*fl*^ mice have relatively normal ERG response in scotopic conditions compared to controls. Raw electroretinogram waveforms from Lsd1^*fl*/*fl*^ controls and Rho-iCre75 Lsd1^*fl*/*fl*^ in scotopic conditions **(A)** after a 10 cd s/m^2^ light flash. At multiple flash intensities, the a-wave **(B)** and b-wave **(C)** for the Rho-iCre75 Lsd1^*fl*/*fl*^ are virtually identical to Lsd1^*fl*/*fl*^ controls. Additionally, there were no statistical differences in the scotopic c-wave between the two groups **(D)**. Two-Way ANOVA with Tukey’s multiple comparisons test. Samples sizes: Lsd1^*fl*/*fl*^ (*n* = 7), Rho-iCre75 Lsd1^*fl*/*fl*^ (*n* = 7).

Supplementary Figure 4 Rho-iCre75 Lsd1^*fl*/*fl*^ animals show no obvious *in vivo* morphological differences compared to controls. In both fundus photos **(A,B)** and SD-OCT images **(C,D)**, there are no signs of obvious differences between the Rho-iCre75 Lsd1^*fl*/*fl*^ and the Lsd1^*fl*/*fl*^ group. Samples sizes: Lsd1^*fl*/*fl*^ (*n* = 5), Rho-iCre75 Lsd1^*fl*/*fl*^ (*n* = 5).

Supplementary Figure 5 Rho-iCre75 Lsd1^*fl*/*fl*^ animals show no post-mortem obvious morphological differences compared to controls. In both whole eye **(A,B)** and magnified retina images **(C,D)**, there are no signs of obvious differences between the Rho-iCre75 Lsd1^*fl*/*fl*^ and the Lsd1^*fl*/*fl*^ control group. Additionally, there is no statistical difference in the ONL cell nuclei counts between the two groups. Two–Way ANOVA with Tukey’s multiple comparisons test. Samples sizes: Lsd1^*fl*/*fl*^ (*n* = 5), Rho-iCre75 Lsd1^*fl*/*fl*^ (*n* = 5).
