# Supplementary material for: Deletion of histone demethylase Lsd1 (Kdm1a) during retinal development leads to defects in retinal function and structure
Source: Front Cell Neurosci. 2023 Feb 10;17:1104592. doi: 10.3389/fncel.2023.1104592 (PMC9950115; doi:10.3389/fncel.2023.1104592)
Supplement: Supplementary file 1 [file Table_1.DOCX]

Supplementary Table 1 – One-Way ANOVA with Tukey’s multiple comparisons test for LSD1 protein levels

| **Tukey’s multiple comparisons test for LSD1 protein levels** | **Adjusted P Value** | **Significant?** | **Summary** | **Mean Diff.** | **95.00% CI of diff.** |
| --- | --- | --- | --- | --- | --- |
| Chx10-Cre Control vs. LSD1^fl/fl^ Control | 0.0486 | Yes | * | -0.2180 | -0.4345 to -0.001438 |
| Chx10-Cre Control vs. Chx10-Cre LSD1^fl/fl^ | 0.0003 | Yes | *** | 0.5098 | 0.2932 to 0.7263 |
| Lsd1^fl/fl^ Control vs. Chx10-Cre LSD1^fl/fl^ | <0.0001 | Yes | **** | 0.7278 | 0.5112 to 0.9443 |

* represents p value <0.05; ** represents p value <0.01; *** represents p value <0.001; **** represents p value <0.0001

Supplementary Table 2 – Two-Way ANOVA with Tukey’s multiple comparisons test for Scotopic A Wave

| **Tukey’s multiple comparisons test for Scotopic A Wave** | **Adjusted P Value** | **Significant?** | **Summary** | **Mean Diff.** | **95.00% CI of diff.** |
| --- | --- | --- | --- | --- | --- |
| **0.001** |  |  |  |  |  |
| Chx10-Cre Control vs. LSD1^fl/fl^ Control | 0.8672 | No | ns | 6.321 | -23.35 to 35.99 |
| Chx10-Cre Control vs. Chx10-Cre LSD1^fl/fl^ | 0.9974 | No | ns | 0.8579 | -28.81 to 30.53 |
| LSD1^fl/fl^ Control vs. Chx10-Cre LSD1^fl/fl^ | 0.9069 | No | ns | -5.464 | -36.45 to 25.52 |
| **0.005** |  |  |  |  |  |
| Chx10-Cre Control vs. LSD1^fl/fl^ Control | 0.9461 | No | ns | 3.937 | -25.73 to 33.61 |
| Chx10-Cre Control vs. Chx10-Cre LSD1^fl/fl^ | 0.8784 | No | ns | 6.030 | -23.64 to 35.70 |
| LSD1^fl/fl^ Control vs. Chx10-Cre LSD1^fl/fl^ | 0.9858 | No | ns | 2.093 | -28.90 to 33.08 |
| **0.01** |  |  |  |  |  |
| Chx10-Cre Control vs. LSD1^fl/fl^ Control | 0.9972 | No | ns | 0.8794 | -28.79 to 30.55 |
| Chx10-Cre Control vs. Chx10-Cre LSD1^fl/fl^ | 0.9976 | No | ns | -0.8154 | -30.48 to 28.85 |
| LSD1^fl/fl^ Control vs. Chx10-Cre LSD1^fl/fl^ | 0.9906 | No | ns | -1.695 | -32.68 to 29.29 |
| **0.1** |  |  |  |  |  |
| Chx10-Cre Control vs. LSD1^fl/fl^ Control | 0.6789 | No | ns | 10.44 | -19.23 to 40.11 |
| Chx10-Cre Control vs. Chx10-Cre LSD1^fl/fl^ | <0.0001 | Yes | **** | 84.00 | 54.33 to 113.7 |
| LSD1^fl/fl^ Control vs. Chx10-Cre LSD1^fl/fl^ | <0.0001 | Yes | **** | 73.56 | 42.57 to 104.5 |
| **1** |  |  |  |  |  |
| Chx10-Cre Control vs. LSD1^fl/fl^ Control | 0.0795 | No | ns | 27.18 | -2.492 to 56.85 |
| Chx10-Cre Control vs. Chx10-Cre LSD1^fl/fl^ | <0.0001 | Yes | **** | 201.1 | 171.4 to 230.8 |
| LSD1^fl/fl^ Control vs. Chx10-Cre LSD1^fl/fl^ | <0.0001 | Yes | **** | 173.9 | 142.9 to 204.9 |
| **10** |  |  |  |  |  |
| Chx10-Cre Control vs. LSD1^fl/fl^ Control | 0.0095 | Yes | ** | 37.48 | 7.809 to 67.15 |
| Chx10-Cre Control vs. Chx10-Cre LSD1^fl/fl^ | <0.0001 | Yes | **** | 285.8 | 256.1 to 315.5 |
| LSD1^fl/fl^ Control vs. Chx10-Cre LSD1^fl/fl^ | <0.0001 | Yes | **** | 248.3 | 217.3 to 279.3 |

* represents p value <0.05; ** represents p value <0.01; *** represents p value <0.001; **** represents p value <0.0001

Supplementary Table 3– Two-Way ANOVA with Tukey’s multiple comparisons test for Scotopic B Wave

| **Tukey’s multiple comparisons test for Scotopic A Wave** | **Adjusted P Value** | **Significant?** | **Summary** | **Mean Diff.** | **95.00% CI of diff.** |
| --- | --- | --- | --- | --- | --- |
| **0.001** |  |  |  |  |  |
| Chx10-Cre Control vs. LSD1^fl/fl^ Control | 0.9436 | No | ns | -10.80 | -90.25 to 68.66 |
| Chx10-Cre Control vs. Chx10-Cre LSD1^fl/fl^ | >0.9999 | No | ns | 0.08773 | -79.37 to 79.54 |
| LSD1^fl/fl^ Control vs. Chx10-Cre LSD1^fl/fl^ | 0.9473 | No | ns | 10.89 | -72.10 to 93.88 |
| **0.005** |  |  |  |  |  |
| Chx10-Cre Control vs. LSD1^fl/fl^ Control | 0.0075 | Yes | ** | 103.1 | 23.65 to 182.6 |
| Chx10-Cre Control vs. Chx10-Cre LSD1^fl/fl^ | <0.0001 | Yes | **** | 286.8 | 207.3 to 366.2 |
| LSD1^fl/fl^ Control vs. Chx10-Cre LSD1^fl/fl^ | <0.0001 | Yes | **** | 183.7 | 100.7 to 266.7 |
| **0.01** |  |  |  |  |  |
| Chx10-Cre Control vs. LSD1^fl/fl^ Control | 0.0046 | Yes | ** | 108.6 | 29.17 to 188.1 |
| Chx10-Cre Control vs. Chx10-Cre LSD1^fl/fl^ | <0.0001 | Yes | **** | 355.3 | 275.9 to 434.8 |
| LSD1^fl/fl^ Control vs. Chx10-Cre LSD1^fl/fl^ | <0.0001 | Yes | **** | 246.7 | 163.7 to 329.7 |
| **0.1** |  |  |  |  |  |
| Chx10-Cre Control vs. LSD1^fl/fl^ Control | 0.0005 | Yes | *** | 131.8 | 52.34 to 211.3 |
| Chx10-Cre Control vs. Chx10-Cre LSD1^fl/fl^ | <0.0001 | Yes | **** | 423.8 | 344.3 to 503.2 |
| LSD1^fl/fl^ Control vs. Chx10-Cre LSD1^fl/fl^ | <0.0001 | Yes | **** | 292.0 | 209.0 to 374.9 |
| **1** |  |  |  |  |  |
| Chx10-Cre Control vs. LSD1^fl/fl^ Control | <0.0001 | Yes | **** | 174.6 | 95.13 to 254.0 |
| Chx10-Cre Control vs. Chx10-Cre LSD1^fl/fl^ | <0.0001 | Yes | **** | 569.4 | 489.9 to 648.8 |
| LSD1^fl/fl^ Control vs. Chx10-Cre LSD1^fl/fl^ | <0.0001 | Yes | **** | 394.8 | 311.8 to 477.8 |
| **10** |  |  |  |  |  |
| Chx10-Cre Control vs. LSD1^fl/fl^ Control | <0.0001 | Yes | **** | 211.4 | 131.9 to 290.8 |
| Chx10-Cre Control vs. Chx10-Cre LSD1^fl/fl^ | <0.0001 | Yes | **** | 690.2 | 610.8 to 769.7 |
| LSD1^fl/fl^ Control vs. Chx10-Cre LSD1^fl/fl^ | <0.0001 | Yes | **** | 478.9 | 395.9 to 561.9 |

* represents p value <0.05; ** represents p value <0.01; *** represents p value <0.001; **** represents p value <0.0001

Supplementary Table 4– One-Way ANOVA with Tukey’s multiple comparisons test for Scotopic C Wave

| **Tukey’s multiple comparisons test for Scotopic C Wave** | **Adjusted P Value** | **Significant?** | **Summary** | **Mean Diff.** | **95.00% CI of diff.** |
| --- | --- | --- | --- | --- | --- |
| Chx10-Cre Control vs. LSD1^fl/fl^ Control | <0.0001 | Yes | **** | 261137 | 166502 to 355773 |
| Chx10-Cre Control vs. Chx10-Cre LSD1^fl/fl^ | <0.0001 | Yes | **** | 457486 | 362851 to 552121 |
| LSD1^fl/fl^ Control vs. Chx10-Cre LSD1^fl/fl^ | .0004 | Yes | *** | 196349 | 97506 to 295192 |

* represents p value <0.05; ** represents p value <0.01; *** represents p value <0.001; **** represents p value <0.0001

Supplementary Table 5– One-Way ANOVA with Tukey’s multiple comparisons test for SD-OCT: Retina Thickness

| **Tukey’s multiple comparisons test for MicronIV: Retina Thickness** | **Adjusted P Value** | **Significant?** | **Summary** | **Mean Diff.** | **95.00% CI of diff.** |
| --- | --- | --- | --- | --- | --- |
| Chx10-Cre Control vs. LSD1^fl/fl^ Control | 0.0217 | Yes | * | 14.35 | 1.920 to 26.78 |
| Chx10-Cre Control vs. Chx10-Cre LSD1^fl/fl^ | <0.0001 | Yes | **** | 59.05 | 47.23 to 70.88 |
| LSD1^fl/fl^ Control vs. Chx10-Cre LSD1^fl/fl^ | <0.0001 | Yes | **** | 44.70 | 30.87 to 58.54 |

* represents p value <0.05; ** represents p value <0.01; *** represents p value <0.001; **** represents p value <0.0001

Supplementary Table 6 – One-Way ANOVA with Tukey’s multiple comparisons test for SD-OCT: ONL Thickness

| **Tukey’s multiple comparisons test for MicronIV: ONL Thickness** | **Adjusted P Value** | **Significant?** | **Summary** | **Mean Diff.** | **95.00% CI of diff.** |
| --- | --- | --- | --- | --- | --- |
| Chx10-Cre Control vs. LSD1^fl/fl^ Control | 0.0731 | No | ns | 4.958 | -0.3980 to 10.31 |
| Chx10-Cre Control vs. Chx10-Cre LSD1^fl/fl^ | <0.0001 | Yes | **** | 23.56 | 18.46 to 28.65 |
| LSD1^fl/fl^ Control vs. Chx10-Cre LSD1^fl/fl^ | <0.0001 | Yes | **** | 18.60 | 12.64 to 24.56 |

* represents p value <0.05; ** represents p value <0.01; *** represents p value <0.001; **** represents p value <0.0001

Supplementary Table 7 – Two-Way ANOVA with Tukey’s multiple comparisons test for ONL counts

| **Tukey’s multiple comparisons test for ONL Counts** | **Adjusted P Value** | **Significant?** | **Summary** | **Mean Diff.** | **95.00% CI of diff.** |
| --- | --- | --- | --- | --- | --- |
| **-1750** |  |  |  |  |  |
| Chx10-Cre Control vs. LSD1^fl/fl^ Control | 0.4692 | No | ns | -24.29 | -81.79 to 33.21 |
| Chx10-Cre Control vs. Chx10-Cre LSD1^fl/fl^ | 0.9169 | No | ns | 6.667 | -49.54 to 62.88 |
| LSD1^fl/fl^ Control vs. Chx10-Cre LSD1^fl/fl^ | 0.1439 | No | ns | 30.95 | -9.980 to 71.88 |
| **-1250** |  |  |  |  |  |
| Chx10-Cre Control vs. LSD1^fl/fl^ Control | 0.9420 | No | ns | 6.200 | -45.28 to 57.68 |
| Chx10-Cre Control vs. Chx10-Cre LSD1^fl/fl^ | 0.0306 | Yes | * | 48.37 | 5.236 to 91.50 |
| LSD1^fl/fl^ Control vs. Chx10-Cre LSD1^fl/fl^ | 0.0697 | No | ns | 42.17 | -3.436 to 87.77 |
| **-750** |  |  |  |  |  |
| Chx10-Cre Control vs. LSD1^fl/fl^ Control | 0.2557 | No | ns | 23.11 | -15.32 to 61.55 |
| Chx10-Cre Control vs. Chx10-Cre LSD1^fl/fl^ | <0.0001 | Yes | **** | 73.07 | 49.08 to 97.06 |
| LSD1^fl/fl^ Control vs. Chx10-Cre LSD1^fl/fl^ | 0.0155 | Yes | * | 49.95 | 10.58 to 89.32 |
| **-250** |  |  |  |  |  |
| Chx10-Cre Control vs. LSD1^fl/fl^ Control | 0.5630 | No | ns | 24.17 | -42.74 to 91.08 |
| Chx10-Cre Control vs. Chx10-Cre LSD1^fl/fl^ | 0.0412 | Yes | * | 56.77 | 2.716 to 110.8 |
| LSD1^fl/fl^ Control vs. Chx10-Cre LSD1^fl/fl^ | 0.4862 | No | ns | 32.60 | -42.00 to 107.2 |
| **0** |  |  |  |  |  |
| Chx10-Cre Control vs. LSD1^fl/fl^ Control |  |  |  |  |  |
| Chx10-Cre Control vs. Chx10-Cre LSD1^fl/fl^ |  |  |  |  |  |
| LSD1^fl/fl^ Control vs. Chx10-Cre LSD1^fl/fl^ |  |  |  |  |  |
| **250** |  |  |  |  |  |
| Chx10-Cre Control vs. LSD1^fl/fl^ Control | 0.8227 | No | ns | 8.429 | -30.40 to 47.26 |
| Chx10-Cre Control vs. Chx10-Cre LSD1^fl/fl^ | 0.0012 | Yes | ** | 52.50 | 26.44 to 78.56 |
| LSD1^fl/fl^ Control vs. Chx10-Cre LSD1^fl/fl^ | 0.0228 | Yes | * | 44.07 | 6.972 to 81.17 |
| **750** |  |  |  |  |  |
| Chx10-Cre Control vs. LSD1^fl/fl^ Control | 0.4584 | No | ns | 10.60 | -13.99 to 35.19 |
| Chx10-Cre Control vs. Chx10-Cre LSD1^fl/fl^ | 0.0004 | Yes | *** | 72.60 | 45.75 to 99.45 |
| LSD1^fl/fl^ Control vs. Chx10-Cre LSD1^fl/fl^ | 0.0006 | Yes | *** | 62.00 | 30.73 to 93.27 |
| **1250** |  |  |  |  |  |
| Chx10-Cre Control vs. LSD1^fl/fl^ Control | 0.8476 | No | ns | -6.486 | -39.12 to 26.15 |
| Chx10-Cre Control vs. Chx10-Cre LSD1^fl/fl^ | 0.0136 | Yes | * | 49.13 | 11.42 to 86.85 |
| LSD1^fl/fl^ Control vs. Chx10-Cre LSD1^fl/fl^ | 0.0044 | Yes | ** | 55.62 | 19.78 to 91.45 |
| **1750** |  |  |  |  |  |
| Chx10-Cre Control vs. LSD1^fl/fl^ Control | 0.9580 | No | ns | 4.457 | -41.83 to 50.75 |
| Chx10-Cre Control vs. Chx10-Cre LSD1^fl/fl^ | 0.1618 | No | ns | 36.27 | -13.91 to 86.45 |
| LSD1^fl/fl^ Control vs. Chx10-Cre LSD1^fl/fl^ | 0.1332 | No | ns | 31.81 | -9.231 to 72.85 |

* represents p value <0.05; ** represents p value <0.01; *** represents p value <0.001; **** represents p value <0.0001

Supplementary Table 8 – Two-Way ANOVA with Tukey’s multiple comparisons test for INL counts

| **Tukey’s multiple comparisons test for INL Counts** | **Adjusted P Value** | **Significant?** | **Summary** | **Mean Diff.** | **95.00% CI of diff.** |
| --- | --- | --- | --- | --- | --- |
| **-1750** |  |  |  |  |  |
| Chx10-Cre Control vs. LSD1^fl/fl^ Control | 0.5925 | No | ns | 10.25 | -20.51 to 41.01 |
| Chx10-Cre Control vs. Chx10-Cre LSD1^fl/fl^ | 0.1471 | No | ns | 22.92 | -8.571 to 54.40 |
| LSD1^fl/fl^ Control vs. Chx10-Cre LSD1^fl/fl^ | 0.3569 | No | ns | 12.67 | -11.25 to 36.58 |
| **-1250** |  |  |  |  |  |
| Chx10-Cre Control vs. LSD1^fl/fl^ Control | 0.2586 | No | ns | 34.74 | -27.52 to 97.01 |
| Chx10-Cre Control vs. Chx10-Cre LSD1^fl/fl^ | 0.0582 | No | ns | 59.60 | -2.654 to 121.9 |
| LSD1^fl/fl^ Control vs. Chx10-Cre LSD1^fl/fl^ | 0.0606 | No | ns | 24.86 | -1.089 to 50.80 |
| **-750** |  |  |  |  |  |
| Chx10-Cre Control vs. LSD1^fl/fl^ Control | 0.3333 | No | ns | 25.91 | -27.03 to 78.85 |
| Chx10-Cre Control vs. Chx10-Cre LSD1^fl/fl^ | 0.0429 | Yes | * | 55.03 | 2.231 to 107.8 |
| LSD1^fl/fl^ Control vs. Chx10-Cre LSD1^fl/fl^ | 0.0204 | Yes | * | 29.12 | 4.808 to 53.43 |
| **-250** |  |  |  |  |  |
| Chx10-Cre Control vs. LSD1^fl/fl^ Control | 0.3259 | No | ns | 22.49 | -19.29 to 64.26 |
| Chx10-Cre Control vs. Chx10-Cre LSD1^fl/fl^ | 0.0322 | Yes | * | 45.20 | 4.984 to 85.42 |
| LSD1^fl/fl^ Control vs. Chx10-Cre LSD1^fl/fl^ | 0.1156 | No | ns | 22.71 | -5.424 to 50.85 |
| **0** |  |  |  |  |  |
| Chx10-Cre Control vs. LSD1^fl/fl^ Control |  |  |  |  |  |
| Chx10-Cre Control vs. Chx10-Cre LSD1^fl/fl^ |  |  |  |  |  |
| LSD1^fl/fl^ Control vs. Chx10-Cre LSD1^fl/fl^ |  |  |  |  |  |
| **250** |  |  |  |  |  |
| Chx10-Cre Control vs. LSD1^fl/fl^ Control | 0.5750 | No | ns | 9.657 | -16.11 to 35.43 |
| Chx10-Cre Control vs. Chx10-Cre LSD1^fl/fl^ | 0.0022 | Yes | ** | 45.63 | 23.25 to 68.01 |
| LSD1^fl/fl^ Control vs. Chx10-Cre LSD1^fl/fl^ | 0.0028 | Yes | ** | 35.98 | 15.13 to 56.82 |
| **750** |  |  |  |  |  |
| Chx10-Cre Control vs. LSD1^fl/fl^ Control | 0.1202 | No | ns | 16.69 | -4.319 to 37.69 |
| Chx10-Cre Control vs. Chx10-Cre LSD1^fl/fl^ | 0.0022 | Yes | ** | 50.40 | 22.10 to 78.70 |
| LSD1^fl/fl^ Control vs. Chx10-Cre LSD1^fl/fl^ | 0.0178 | Yes | * | 33.71 | 6.716 to 60.71 |
| **1250** |  |  |  |  |  |
| Chx10-Cre Control vs. LSD1^fl/fl^ Control | 0.0333 | Yes | * | 16.11 | 1.336 to 30.89 |
| Chx10-Cre Control vs. Chx10-Cre LSD1^fl/fl^ | 0.0124 | Yes | * | 34.23 | 9.222 to 59.24 |
| LSD1^fl/fl^ Control vs. Chx10-Cre LSD1^fl/fl^ | 0.1612 | No | ns | 18.12 | -7.121 to 43.36 |
| **1750** |  |  |  |  |  |
| Chx10-Cre Control vs. LSD1^fl/fl^ Control | 0.0209 | Yes | * | 23.03 | 4.100 to 41.96 |
| Chx10-Cre Control vs. Chx10-Cre LSD1^fl/fl^ | 0.0476 | Yes | * | 30.43 | 0.3557 to 60.51 |
| LSD1^fl/fl^ Control vs. Chx10-Cre LSD1^fl/fl^ | 0.7371 | No | ns | 7.405 | -21.64 to 36.45 |

* represents p value <0.05; ** represents p value <0.01; *** represents p value <0.001; **** represents p value <0.0001

Supplementary Table 9 – Two-Way ANOVA with Tukey’s multiple comparisons test for RGCL counts

| **Tukey’s multiple comparisons test for RGCL Counts** | **Adjusted P Value** | **Significant?** | **Summary** | **Mean Diff.** | **95.00% CI of diff.** |
| --- | --- | --- | --- | --- | --- |
| **-1750** |  |  |  |  |  |
| Chx10-Cre Control vs. LSD1^fl/fl^ Control | 0.0089 | Yes | ** | -7.083 | -12.05 to -2.112 |
| Chx10-Cre Control vs. Chx10-Cre LSD1^fl/fl^ | 0.1684 | No | ns | -3.119 | -7.626 to 1.388 |
| LSD1^fl/fl^ Control vs. Chx10-Cre LSD1^fl/fl^ | 0.0467 | Yes | * | 3.964 | 0.08016 to 7.848 |
| **-1250** |  |  |  |  |  |
| Chx10-Cre Control vs. LSD1^fl/fl^ Control | 0.2694 | No | ns | -6.667 | -18.52 to 5.190 |
| Chx10-Cre Control vs. Chx10-Cre LSD1^fl/fl^ | 0.4656 | No | ns | -2.238 | -7.664 to 3.188 |
| LSD1^fl/fl^ Control vs. Chx10-Cre LSD1^fl/fl^ | 0.4788 | No | ns | 4.429 | -7.737 to 16.59 |
| **-750** |  |  |  |  |  |
| Chx10-Cre Control vs. LSD1^fl/fl^ Control | 0.1135 | No | ns | -5.500 | -12.54 to 1.542 |
| Chx10-Cre Control vs. Chx10-Cre LSD1^fl/fl^ | 0.9988 | No | ns | -0.07143 | -4.153 to 4.010 |
| LSD1^fl/fl^ Control vs. Chx10-Cre LSD1^fl/fl^ | 0.1271 | No | ns | 5.429 | -1.650 to 12.51 |
| **-250** |  |  |  |  |  |
| Chx10-Cre Control vs. LSD1^fl/fl^ Control | 0.6711 | No | ns | -2.167 | -9.253 to 4.920 |
| Chx10-Cre Control vs. Chx10-Cre LSD1^fl/fl^ | 0.9436 | No | ns | 0.5476 | -4.414 to 5.509 |
| LSD1^fl/fl^ Control vs. Chx10-Cre LSD1^fl/fl^ | 0.4473 | No | ns | 2.714 | -4.012 to 9.440 |
| **0** |  |  |  |  |  |
| Chx10-Cre Control vs. LSD1^fl/fl^ Control |  |  |  |  |  |
| Chx10-Cre Control vs. Chx10-Cre LSD1^fl/fl^ |  |  |  |  |  |
| LSD1^fl/fl^ Control vs. Chx10-Cre LSD1^fl/fl^ |  |  |  |  |  |
| **250** |  |  |  |  |  |
| Chx10-Cre Control vs. LSD1^fl/fl^ Control | 0.1638 | No | ns | -3.767 | -8.984 to 1.450 |
| Chx10-Cre Control vs. Chx10-Cre LSD1^fl/fl^ | 0.2351 | No | ns | -2.881 | -7.520 to 1.759 |
| LSD1^fl/fl^ Control vs. Chx10-Cre LSD1^fl/fl^ | 0.8124 | No | ns | 0.8857 | -3.303 to 5.075 |
| **750** |  |  |  |  |  |
| Chx10-Cre Control vs. LSD1^fl/fl^ Control | 0.2925 | No | ns | -3.333 | -9.134 to 2.467 |
| Chx10-Cre Control vs. Chx10-Cre LSD1^fl/fl^ | 0.7786 | No | ns | -1.190 | -6.308 to 3.927 |
| LSD1^fl/fl^ Control vs. Chx10-Cre LSD1^fl/fl^ | 0.3907 | No | ns | 2.143 | -2.464 to 6.750 |
| **1250** |  |  |  |  |  |
| Chx10-Cre Control vs. LSD1^fl/fl^ Control | 0.0606 | No | ns | -3.200 | -6.564 to 0.1638 |
| Chx10-Cre Control vs. Chx10-Cre LSD1^fl/fl^ | 0.0720 | No | ns | -1.857 | -3.880 to 0.1653 |
| LSD1^fl/fl^ Control vs. Chx10-Cre LSD1^fl/fl^ | 0.4705 | No | ns | 1.343 | -1.982 to 4.667 |
| **1750** |  |  |  |  |  |
| Chx10-Cre Control vs. LSD1^fl/fl^ Control | 0.0011 | Yes | ** | -6.733 | -10.03 to -3.432 |
| Chx10-Cre Control vs. Chx10-Cre LSD1^fl/fl^ | 0.0107 | Yes | * | -4.190 | -7.322 to -1.059 |
| LSD1^fl/fl^ Control vs. Chx10-Cre LSD1^fl/fl^ | 0.1827 | No | ns | 2.543 | -1.093 to 6.179 |

* represents p value <0.05; ** represents p value <0.01; *** represents p value <0.001; **** represents p value <0.0001

Supplementary Table 10 – One-Way ANOVA with Tukey’s multiple comparisons test for Retinal Lengths

| **Tukey’s multiple comparisons test for Retina Lengths** | **Adjusted P Value** | **Significant?** | **Summary** | **Mean Diff.** | **95.00% CI of diff.** |
| --- | --- | --- | --- | --- | --- |
| Chx10-Cre Control vs. LSD1^fl/fl^ Control | 0.9983 | No | ns | 0.005617 | -0.2593 to 0.2706 |
| Chx10-Cre Control vs. Chx10-Cre LSD1^fl/fl^ | 0.0462 | Yes | * | 0.2784 | 0.004396 to 0.5524 |
| LSD1^fl/fl^ Control vs. Chx10-Cre LSD1^fl/fl^ | 0.0330 | Yes | * | 0.2728 | 0.02103 to 0.5245 |

* represents p value <0.05; ** represents p value <0.01; *** represents p value <0.001; **** represents p value <0.0001

Supplementary Table 11 – One-Way ANOVA with Tukey’s multiple comparisons test for Outer Segments

| **Tukey’s multiple comparisons test for MicronIV: ONL Thickness** | **Adjusted P Value** | **Significant?** | **Summary** | **Mean Diff.** | **95.00% CI of diff.** |
| --- | --- | --- | --- | --- | --- |
| Chx10-Cre Control vs. LSD1^fl/fl^ Control | .9998 | No | ns | .04482 | -6.178 to 6.267 |
| Chx10-Cre Control vs. Chx10-Cre LSD1^fl/fl^ | .0015 | Yes | ** | 10.65 | 4.743 to 16.55 |
| LSD1^fl/fl^ Control vs. Chx10-Cre LSD1^fl/fl^ | .0016 | Yes | ** | 10.60 | 4.698 to 16.50 |

* represents p value <0.05; ** represents p value <0.01; *** represents p value <0.001; **** represents p value <0.0001

Supplementary Table 12 – One-Way ANOVA with Tukey’s multiple comparisons test for Inner Segments

| **Tukey’s multiple comparisons test for MicronIV: ONL Thickness** | **Adjusted P Value** | **Significant?** | **Summary** | **Mean Diff.** | **95.00% CI of diff.** |
| --- | --- | --- | --- | --- | --- |
| Chx10-Cre Control vs. LSD1^fl/fl^ Control | .6267 | No | ns | .9919 | -1.936 to 3.920 |
| Chx10-Cre Control vs. Chx10-Cre LSD1^fl/fl^ | .0001 | Yes | *** | 6.880 | 4.308 to 9.451 |
| LSD1^fl/fl^ Control vs. Chx10-Cre LSD1^fl/fl^ | .0006 | Yes | *** | 5.888 | 3.088 to 8.687 |

* represents p value <0.05; ** represents p value <0.01; *** represents p value <0.001; **** represents p value <0.0001
