# Supplementary figures and images for: Deletion of histone demethylase Lsd1 (Kdm1a) during retinal development leads to defects in retinal function and structure
Source: Front Cell Neurosci. 2023 Feb 10;17:1104592. doi: 10.3389/fncel.2023.1104592 (PMC9950115; doi:10.3389/fncel.2023.1104592)

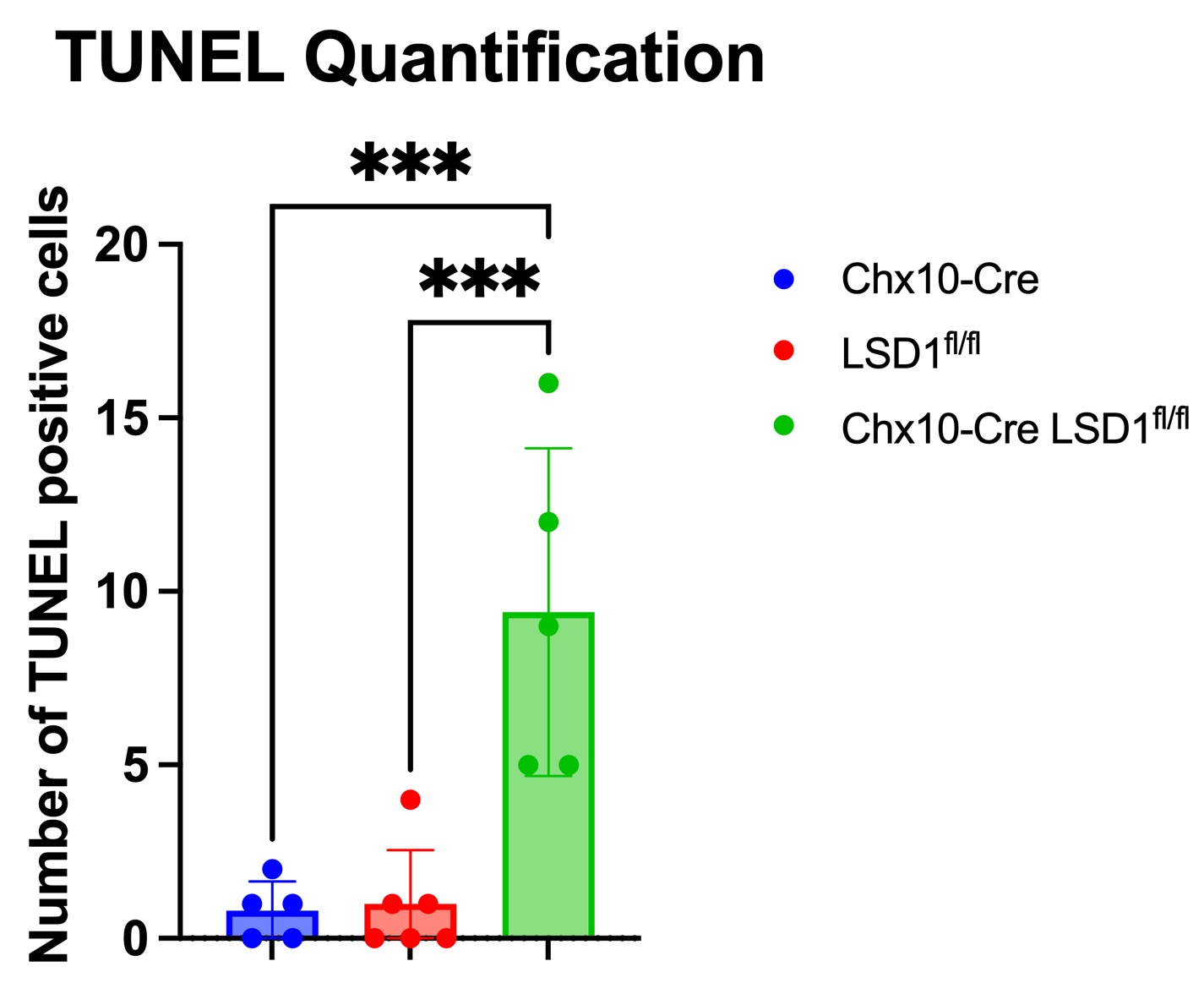

Supplement: Supplementary Figure 1 — Increased number of TUNEL positive cells in the Chx10-Cre Lsd1fl/fl compared to the controls. Two–Way ANOVA with Tukey’s multiple comparisons test. ***Represents p-value < 0.001. Samples sizes: Chx10-Cre (n = 5), Lsd1fl/fl (n = 5), and Chx10-Cre Lsd1fl/fl (n = 5). [file Image_1.jpg]

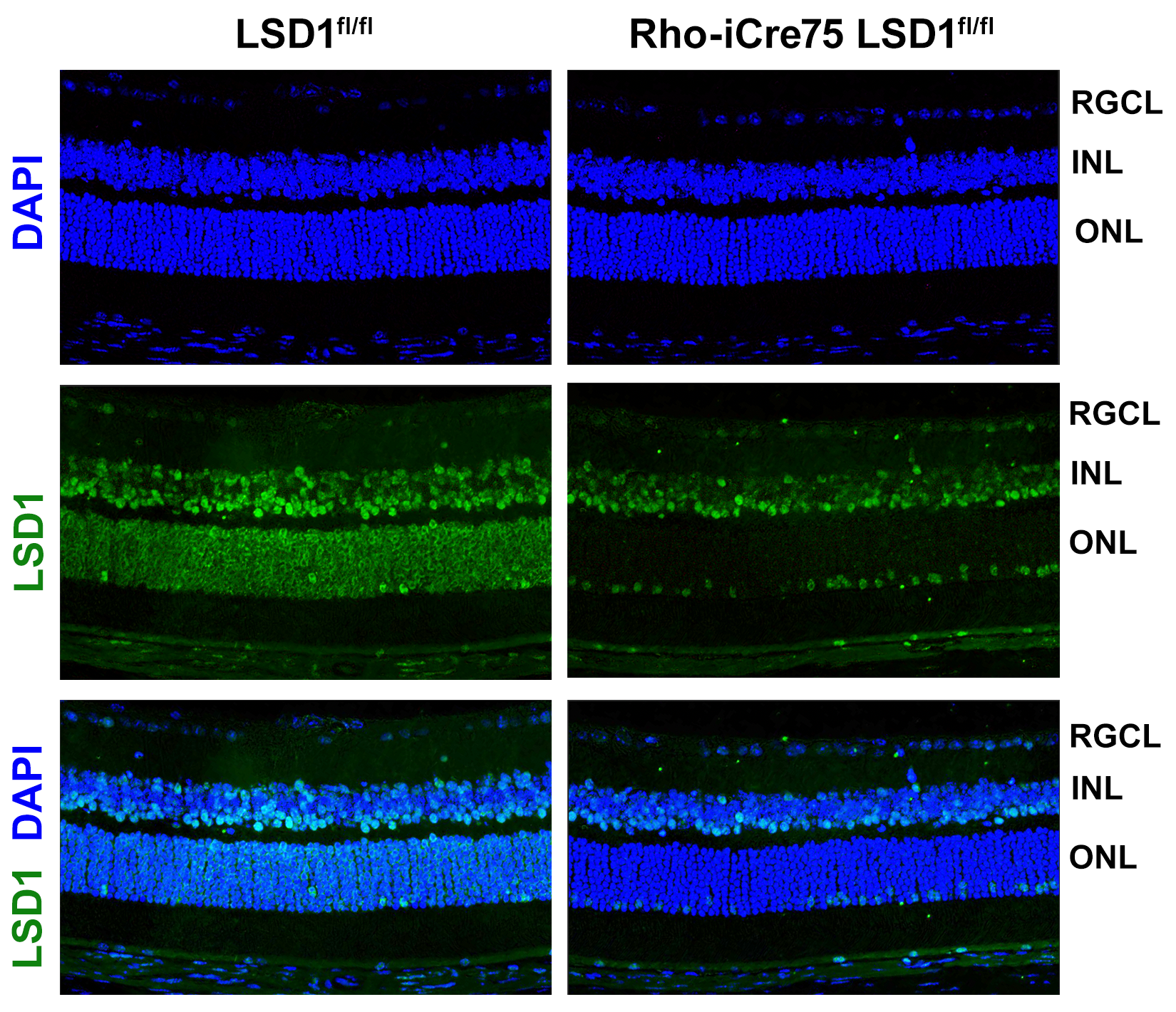

Supplement: Supplementary Figure 2 — Rho-iCre75 Lsd1fl/fl mice have reduced expression of LSD1 compared to Lsd1fl/fl controls. Immunofluorescence staining of LSD1 in P60 retinas showed normal LSD1 protein expression in all major cell types in the Lsd1fl/fl controls; however, there is a reduction in LSD1 specifically in rod photoreceptors, but no other major cell type, in the Rho-iCre75 Lsd1fl/fl mice. Samples sizes: Lsd1fl/fl (n = 3), Rho-iCre75 Lsd1fl/fl (n = 3). [file Image_2.TIF]

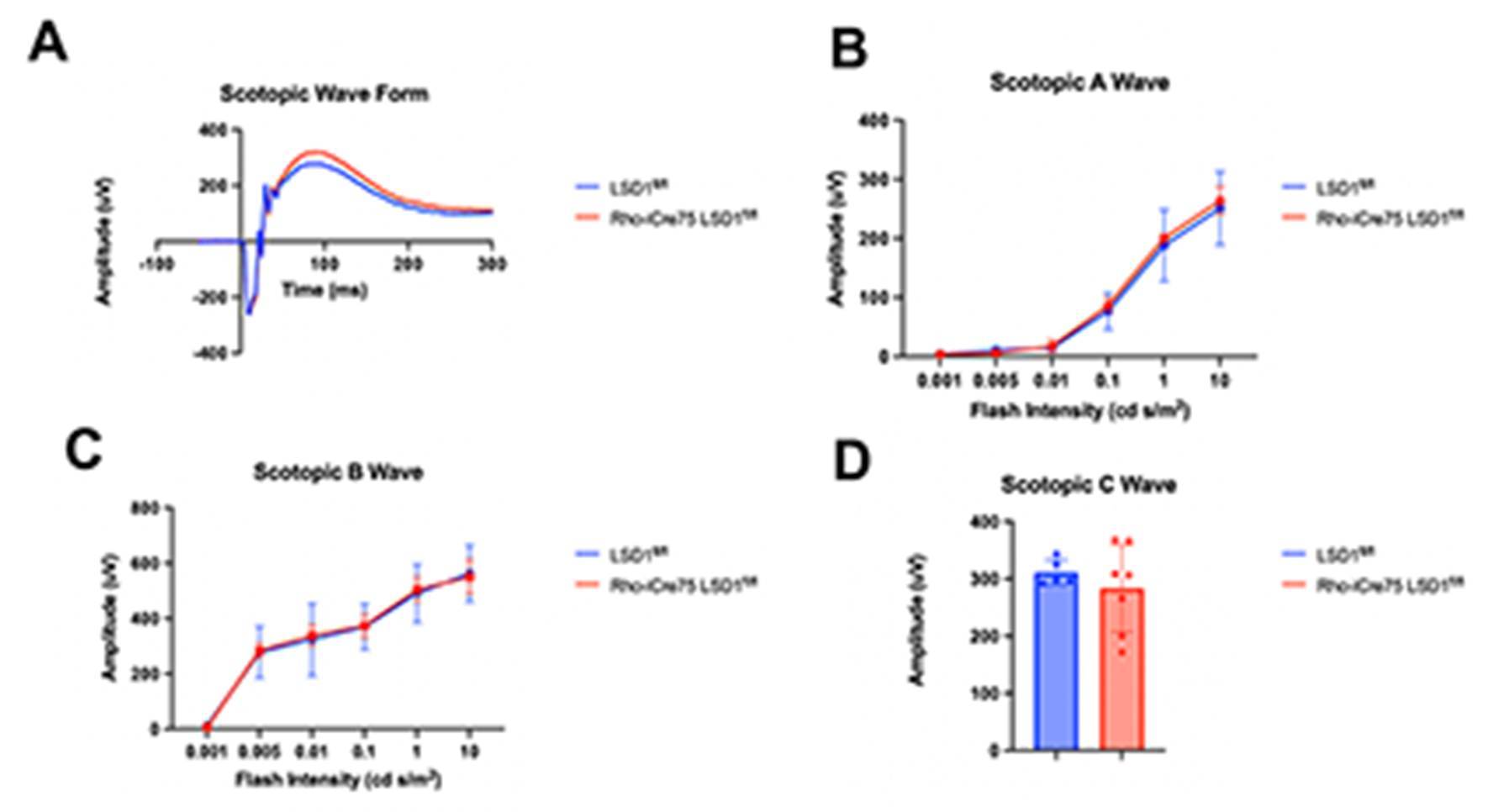

Supplement: Supplementary Figure 3 — Rho-iCre75 Lsd1fl/fl mice have relatively normal ERG response in scotopic conditions compared to controls. Raw electroretinogram waveforms from Lsd1fl/fl controls and Rho-iCre75 Lsd1fl/fl in scotopic conditions (A) after a 10 cd s/m2 light flash. At multiple flash intensities, the a-wave (B) and b-wave (C) for the Rho-iCre75 Lsd1fl/fl are virtually identical to Lsd1fl/fl controls. Additionally, there were no statistical differences in the scotopic c-wave between the two groups (D). Two-Way ANOVA with Tukey’s multiple comparisons test. Samples sizes: Lsd1fl/fl (n = 7), Rho-iCre75 Lsd1fl/fl (n = 7). [file Image_3.TIF]

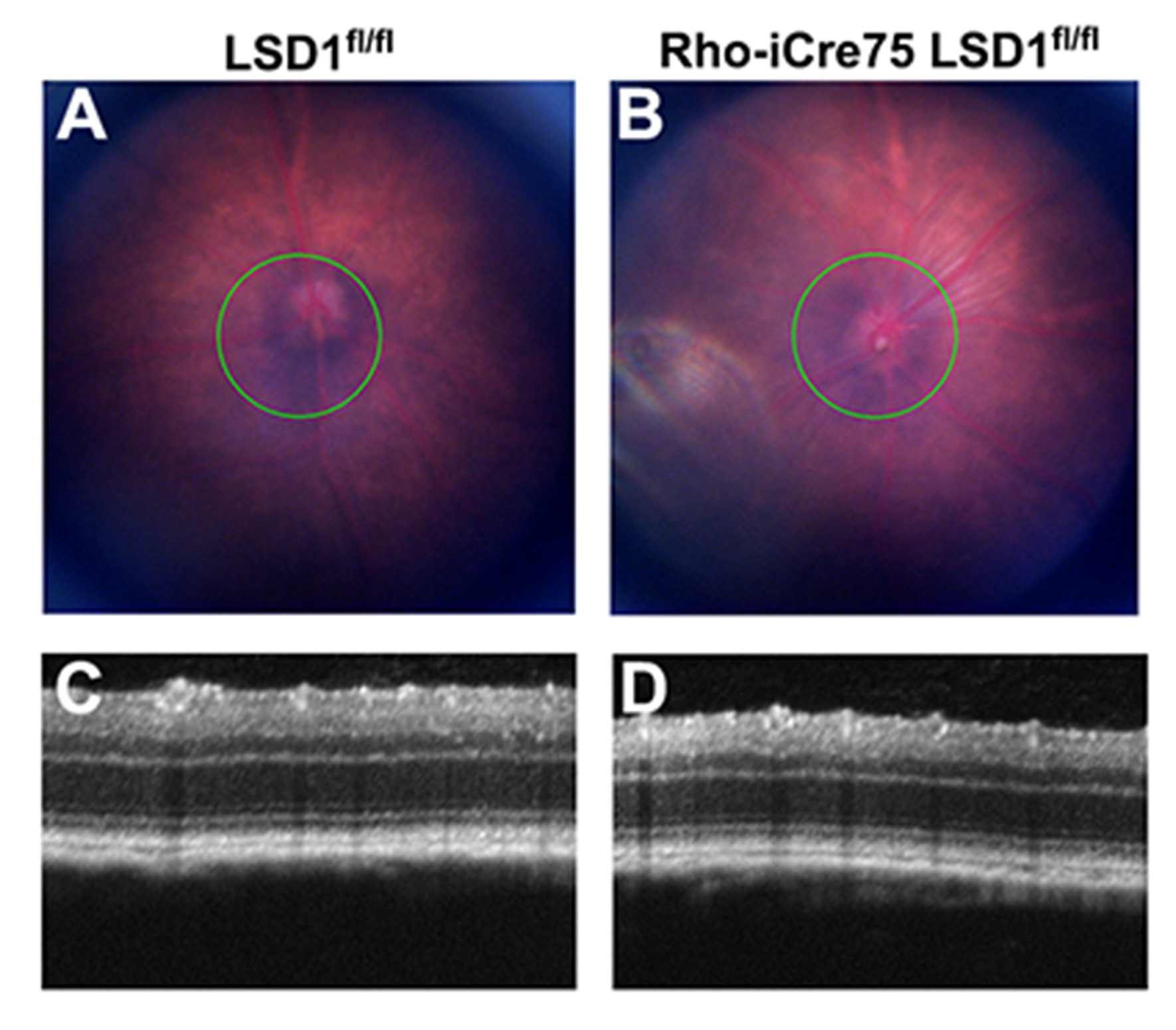

Supplement: Supplementary Figure 4 — Rho-iCre75 Lsd1fl/fl animals show no obvious in vivo morphological differences compared to controls. In both fundus photos (A,B) and SD-OCT images (C,D), there are no signs of obvious differences between the Rho-iCre75 Lsd1fl/fl and the Lsd1fl/fl group. Samples sizes: Lsd1fl/fl (n = 5), Rho-iCre75 Lsd1fl/fl (n = 5). [file Image_4.TIF]

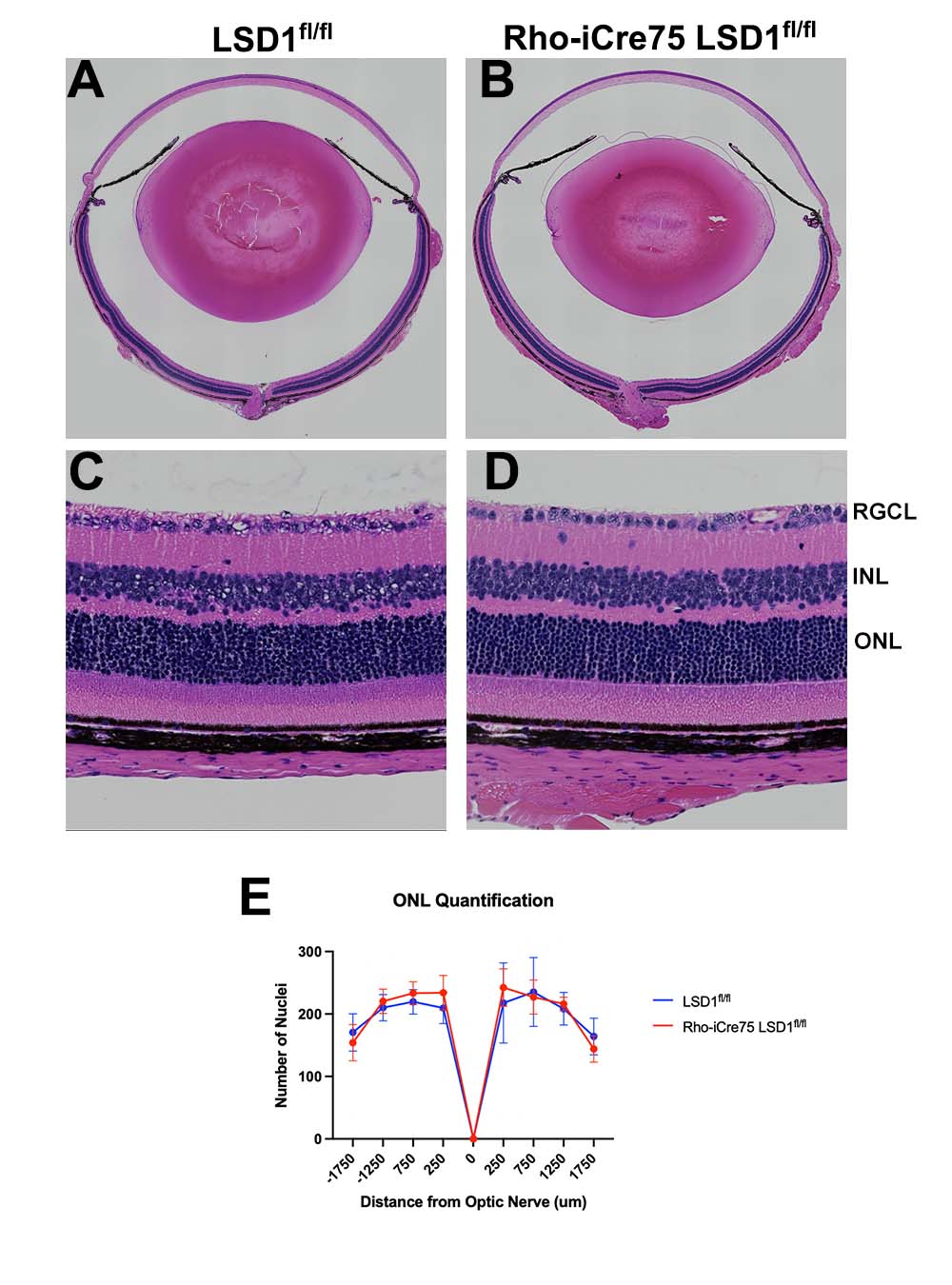

Supplement: Supplementary Figure 5 — Rho-iCre75 Lsd1fl/fl animals show no post-mortem obvious morphological differences compared to controls. In both whole eye (A,B) and magnified retina images (C,D), there are no signs of obvious differences between the Rho-iCre75 Lsd1fl/fl and the Lsd1fl/fl control group. Additionally, there is no statistical difference in the ONL cell nuclei counts between the two groups. Two–Way ANOVA with Tukey’s multiple comparisons test. Samples sizes: Lsd1fl/fl (n = 5), Rho-iCre75 Lsd1fl/fl (n = 5). [file Image_5.jpg]
